# Twenty-Seven Year Response of South Carolina Coastal Plain Forests Affected by Hurricane Hugo

**DOI:** 10.3390/plants12040691

**Published:** 2023-02-04

**Authors:** Reid Heaton, Bo Song, Thomas Williams, William Conner, Zachary Baucom, Brian Williams

**Affiliations:** 1 Belle W. Baruch Institute of Coastal Ecology and Forest Science, Clemson University, P.O. Box 596, Georgetown, SC 29442, USA; 2 South Carolina Governor’s School for Science and Mathematics, 401 Railroad Avenue, Hartsville, SC 29550, USA

**Keywords:** resiliency, disturbance, composition, structure, *Quercus laurifolia* Michx., *Pinus taeda* L., *Taxodium distichum* (L.) Rich., southeastern US

## Abstract

In 1989, Hurricane Hugo inflicted catastrophic damage on approximately 1.8 million ha of forested land in South Carolina. The purpose of this study was to monitor species compositional shifts and structural changes in several forest types following the hurricane’s disturbance. The immediate consequences of hurricane damage are well documented, but there are few studies based on the long-term compositional and structural changes that may result from hurricane disturbance, especially in temperate forest ecosystems. Forty-two forested plots were monitored within four study areas that received varying degrees of hurricane damage. Inventories included species, damage class, tree diameter, and regeneration. The objectives of this study were (1) to compare the recovery speed of wetland forests (e.g., bottomland hardwood swamps and cypress-tupelo swamps) to that of upland pine and hardwood forests; (2) to discover how the degree of hurricane damage can affect the timing and the pattern of forest recovery in the coastal plain; and (3) to compare individual species response patterns across different forest types and at different levels of initial damage. Over the 27-year period following the hurricane, successional pathways have been variable among plots of different forest types and intensity of initial disturbance. We have observed an expected increase in basal area (BA) following the disturbance. Sapling populations in many species have increased dramatically, and some of these populations have begun to thin in recent years. In several forest types, loblolly pine (*Pinus taeda* L.—not a predominant species in these sites prior to the hurricane) responded quickly and overtook some dominant species in BA and tree/sapling abundance. Several other species that were not a major component of the tree strata (wax myrtle [*Morella cerifera* (L.) Small], green ash [*Fraxinus pennsylvanica* Marsh.], and the invasive Chinese tallow [*Triadica sebifera* (L.) Small]) showed a large increase in sapling population. Overall, recovery speed and species resilience were specific to forest types and damage severity. The intensity and frequency of hurricanes may increase in the future as sea surface temperatures rise. Understanding how coastal forests respond to major hurricanes in the short-term and the long-term will aid us in preparing for future hurricanes and for potential changes in disturbance regimes.

## 1. Introduction

**Figure 1:**
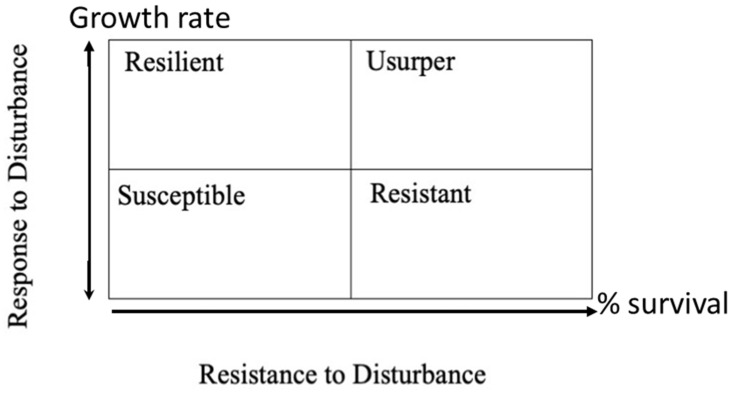
Diagram of categorizations of individual species roles within a forest type after hurricane disturbance (adapted from [18]).

Coastal forests endure frequent hurricanes and suffer effects from high winds, heavy rain, flooding, salt spray, and saltwater intrusion. These forces can drastically alter the composition of a forest in a matter of hours. Trees are broken, uprooted, and defoliated by wind and rain. Trees can also be damaged by salt brought ashore by the wind and soil salinization due to storm surge. The remaining trees are left especially vulnerable to fire and to insect infestation [1,2]. The immediate consequences of hurricane damage are well documented [3,4]. However, there has been less research on the long-term compositional and structural changes that may result from the catastrophic damage [5]. Long-term alterations in forest species composition and structure have been analyzed in tropical ecosystems [6,7], but little information exists on the recovery of temperate forest ecosystems. One of the few studies focusing on long-term dynamics of temperate forests following a hurricane was performed on selected sections of the Harvard Forest after a 1938 hurricane [8,9]. After four growing seasons, a high stem density and low basal area (BA) persisted relative to pre-storm levels. After 46 growing seasons, the BA, density, and dominant species of the forest were beginning to resemble pre-storm conditions. This return to a previous equilibrium ecosystem is consistent with a Clementsian [10] view of succession to a single equilibrium state. The alternative Gleasonian [11] view of succession regards ecosystems as self-organized systems of individual plant species that may have multiple successional pathways and equilibria [12]. Many hurricane studies in tropical and temperate forest ecosystems imply that differences in recovery pathways may be largely influenced by pre-hurricane composition, location in relation to the path of the storm and by local hurricane intensity [12,13,14,15,16,17]. Individual species may be classified within a forest type in terms of resistance and resilience to disturbance based on the interpretations of Bellingham et al. [18] and Batista and Pratt [19]. The possible roles that a species may assume following a disturbance are summarized in Figure 1. The resistance to disturbance and response to disturbance axes can evaluated by the portion of a species that survived and changes in growth rate following the storm [19].

The Gulf and Atlantic coasts of the United States are impacted by several tropical cyclones each year varying in strength from depression (<15 m/s) to major hurricane (>49 m/s), with frequencies determined by the geography of the coast [20]. Hurricane Hugo struck the coast of South Carolina on 23 September 1989, as a major hurricane with maximum winds 60 m/s [21]. As forest products are a major economic driver in South Carolina, research on the direct effects of Hurricane Hugo was intense and has been compiled by Haywood and Hook [22]. Hurricane Hugo inflicted water and wind damage on approximately 1.8 million hectares of forested land in South Carolina, USA [1]. A wind damage assessment of forested land in the path of Hugo was done using the Forest Inventory and Analysis (FIA) plots located across South Carolina [23]. The FIA data was also used to estimate wind speeds associated with the damage assessment [24]. Wind damage assessment was more intensively studied in two forest preserves: Beidler Forest [10] and Congaree National Park [25], and two research forests: Hobcaw Forest [26] and Santee Experimental Forest [1]. Hobcaw Forest was also subject to storm surge and soil salinization [27] that caused continued mortality through 1991 [28].

Although these four intensive study areas varied widely in soils, hydrology, land use history, and hurricane damage, they did provide sites where damage had been assessed, and management of the sites assured an opportunity for long-term studies with minimal anthropogenic influence. In 1991, Charles Gresham designed a series of long-term plots to study hurricane recovery on Hobcaw Forest. With a grant from the Andrew W. Mellon Foundation, he was able to expand that design to include the other three intensively measured sites.

This paper documents the results of 23 years of monitoring long-term forest recovery from Hurricane Hugo on the coastal plain of South Carolina, USA. The original objective of the study was to contrast recovery rate of wetland forest types (e.g., bottomland hardwood swamps and cypress-tupelo swamps) to that of upland pine and hardwood forests as a Clementsian [10] return to the previous stable state. Such a comparison is hampered as forest types are not evenly distributed among sites. The cypress-tupelo cover type is the only type that is present at all study sites.

However, the study sites vary by a variety of factors in addition to Hurricane damage and can be examined in a manner similar to Bellingham et al. [18] to determine if species behave consistently (being resistant, resilient, susceptible, or usurper) across gradients of damage, landscape pattern, soil fertility, and hydrologic inputs.

Original data were collected by a four-letter species code that refers to common names for these species used in southeastern U.S. forestry as used in FIA analysis. In some figures the four-letter code for each species is used to save space. These codes and common names are listed with the taxonomic name of the species in Table 1.

## 2. Materials and Methods

### 2.1. Study Area Site Descriptions

**Figure 2:**
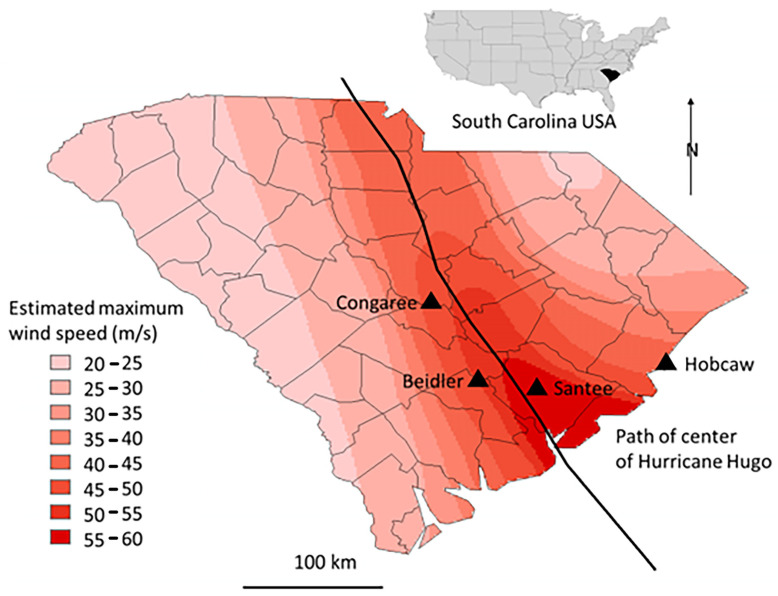
Illustration of the location of the four research sites in relation to: the coast of South Carolina, USA, the path of the eye of Hurricane Hugo [21], and the estimated maximum wind speeds based on damage evaluated on the Fugita scale [24].

Four study areas: Beidler Forest, Congaree National Park, Hobcaw Barony, and Santee Experimental Forest, were established in 1993 (Figure 2) based on five forest cover types [14], and have been monitored on an approximately three-year cycle since then. These study areas received varying degrees and types of damage from Hurricane Hugo. They have been protected from fire and logging and allowed to regenerate naturally since Hurricane Hugo.

(1) The Beidler Forest (Lat. 33°9′ N Long. 80°19′ W) is an approximately 6500 ha forest preserve and instructional forest under the administration of the Nature Conservancy. The forest is located within a depressional area known as Four Hole Swamp. Although shaped like a river floodplain, the depression has no continuous stream but only four areas of open water shaped like ox-bow ponds. Three major wetland forest types are cypress-tupelo stands at the lowest elevations, bottomland hardwood stands in the floodplain areas, and ridge bottom stands that are rarely flooded. The ridge bottom forest type is unique to Beidler Forest (Table 2) and has been analyzed with the upland pine hardwood type as it was the best drained type at Beidler. All three types are located on Bethera soils, a very poorly drained soil with loamy surface soil and clay subsoil. Land elevations vary from 14–16 m.

Hurricane damage assessment [29] found the least damage in the cypress-tupelo community (4% mortality) and greatest in the ridge bottom community (47%) with the bottomland hardwood community intermediate (24%). Greatest mortality was in spruce pine (*Pinus glabra*) (93%) and least in water tupelo (*Nyssa aquatica*) (1%) and blackgum (3%). The Beidler Forest is 69 km from the coast, and 21 km west of the path of the center of Hugo. Maximum wind speeds were estimated at 50 m/s (Figure 2).

(2) Congaree National Park (Lat. 33°49′ N Long. 80°50′ W) contains the largest contiguous tract (approx. 9000 ha) of old-growth bottomland hardwood forest in the United States. The Congaree River floodplain is dominated by bottomland hardwood stands. Cypress-tupelo stands are located in creeks and sloughs, and a pine-hardwood community is located on better drained sections of the floodplain. Bottomland hardwood and pine-hardwood types were located on Tawcaw and Congaree Soil series that are silty clay loams with Congaree being moderately well drained and Tawcaw being somewhat poorly drained. Cypress-tupelo communities were located on Chastain series, poorly drained clay soils. Elevation varied from 26 m to 34 m among the study sites in a less than intuitive way. The highest elevation was a cypress-tupelo slough at 34 m, and the lowest in the bottomland hardwoods. In this active floodplain, small scale topography was more important to vegetation type than absolute elevation.

Hurricane damage assessment [25] also found the least damage was done in the cypress-tupelo type with less than 10% mortality of the dominant cypress and tupelo trees. Uprooting was common for associated sweetgum, green ash, and red maple trees. These same three species were dominant, but less frequently uprooted in the bottomland hardwood type. They also found large loblolly pines were heavily damaged in the bottomland hardwood type. Congaree National Forest is 143 km from the coast, and 24 km west of the center of Hugo. Wind speeds at Congaree National Park were estimated at 45–50 m/s (Figure 2).

(3) Hobcaw Barony (Lat. 33°20′ N Long. 79°12′ W) is a 7100 ha forested area on the southern tip of the Waccamaw peninsula north of Georgetown, SC. The peninsula is a series of geologically young marine terraces. The linear orientation of the landscape produced narrow vegetation types. The cypress-tupelo type is located along a narrow stream edge (<200 m wide) that is less than 2 m in elevation and connected to the North Inlet salt marsh. The upland pine hardwood type is also on a narrow ridge that is also less than 3 m in elevation and located near the edge of the North Inlet marsh. There is no large floodplain on Hobcaw precluding a bottomland hardwood type to be measured. Soils beneath the cypress-tupelo type was Hobcaw, a very poorly drained soil with clay subsoil. Hobcaw soil is similar to Bethera except it has high base saturation making it an alphasol rather than an ultisol. The upland pine hardwood stand is on Leon soil, a deep, poorly drained sandy soil.

Wind damage on Hobcaw was less severe and species survival differences were pronounced [26]. Cypress, swamp tupelo, and live oak showed the least wind damage with 89.5% of cypress, 91.5% of swamp tupelo, and 81.8% of live oak having no or light damage. The highest percentage of severe damage were found for pond pine (42.4%), laurel oak (32.1%), and water oak (34.6%). Loblolly pine severe damage (16.8%) was significantly greater than longleaf pine (11.2%). Hobcaw Barony is 5.5 km from the coast, and 72 km east of the center of Hugo with estimated maximum wind speeds of 40 to 45 m/s (Figure 2).

Saltwater intrusion in North Inlet during Hurricane Hugo covered both types on the Hobcaw Forest. Initially, sea water filled the unsaturated soil of the pine-hardwood type [27]. Subsequent rain moved salt in the water table aquifer to the cypress-tupelo swamp and resulted in subsequent mortality of nearly all species located where subsurface flow resulted in concentration of salt contaminated groundwater by 1992 [28].

(4) The Santee Experimental Forest (Lat. 33°7′ N Long. 79°47′ W) is a section of the Francis Marion National Forest, located north of Charleston, SC. The floodplain of a small creek supports cypress-tupelo and bottomland hardwood forest types. These forest types are separated by an earthen dike built to control water for rice cultivation during the 18th and 19th centuries. Like similar types in the Beidler Forest, these were also on Bethera soil. The pine-hardwood type is located further upslope and was on Wahee, a somewhat poorly drained soil with clay loam subsurface.

The Santee Experimental Forest was the most severely wind impacted site of the comparison. The site was only 14 km east of the center of the storm, and only 28 km from the coast with estimated wind speeds of 55–60 m/s (Figure 2). Damage on the Santee Experimental Forest and the surrounding Francis Marion National Forest did not show species differences, only differences in tree size were important. Trees over 25 cm in diameter at breast height (DBH) approached 90% mortality [1].

### 2.2. Data Collection and Analysis

The sampling plan called for four 0.2 ha sample plots in each vegetation type and each sampling site for a total of 50 plots. However, site limitations resulted in only 42 being studied throughout the entire period (Table 2). Study plots were 20 m × 100 m and were re-measured in 1994, 1997, 2000, 2003, 2007, 2010, 2013, and 2016. Consistent techniques of tree measurement were used throughout the study [14]. All trees (≥2.5 cm in DBH) were permanently numbered, tagged, and DBH measured. Plots were divided into five 20 m × 20 m subplots and marked by aluminum poles to aid in relocating tree numbers. In each plot, all trees ≥ 2.5 cm in DBH were marked with a permanent ID tag at DBH to ensure that data remained consistent throughout the study. Efforts to maintain an accurate 100% tally of all trees in each plot included sequential numbering of trees in each plot starting in the SW corner of the first subplot, a designated tally person with a data sheet from the previous measurement assuring that measurements of the two field technicians included every tree in the subplot, and aluminum tree tags all facing SW that were bent at ninety degrees or straightened during each measurement period. In this way, all trees were measured in the subplot before moving to the next. Ingrowth was marked with new aluminum tags and mortality could often be confirmed by finding the tag on a dead tree. At the end of the study (year 23) all data were examined to find trees that were missed and counted as mortality. Marked trees were identified by species and given a damage class. Data collected during each field season consisted of DBH, current damage class of trees, and small (<2.5 cm DBH) regeneration. Stems with a DBH of 10 cm or greater were analyzed as trees, while stems with a DBH of 2.5 to 9.9 cm were analyzed as saplings.

Basal area (BA) was calculated for each species within each vegetation type at each location. BA per hectare were calculated using the formula:

$$\mathrm{BA}=\left(\left(\Sigma \frac{\pi {\left(\frac{d}{2}\right)}^{2}}{10,000}\right)\times 0.2n\right),$$

where:

BA is basal area in m^2^/ha, *d* is DBH in cm, and *n* is the number of plots recorded (Table 2).

Dominant species for each forest cover type were identified using an Importance Value (IV), based on the 2016 data collection. Importance value was based on both BA and density (stems/ha). IVs were calculated for each species at each site using the formula:

IV = [((Ns/Nt)) + (BAs/BAt))/2)] × 100).

where:

IV—importance value, Ns is number of stems in species, s, Nt is total number of stems, BAs is basal area of species, s, and BAt is total basal area.

The IVs of all species at each site are given in Tables S1–S11 in the Supplementary Data. At each site we used the importance value to determine the number of species that represented 80% of the site total. For the cypress tupelo type six species represented 80% of the importance values. For the bottomland hardwood type, seven species represented 80% and for the upland pine hardwood type, nine species. Those species were then ranked by importance values and used for analysis in an altered Bellingham et al. [18] method.

The Bellingham et al. [18] method evaluates the position of a species in their matrix based on the density and rate of growth in the pre-disturbance forest to the rate of growth of that species in the post-disturbance forest. For this study, we had no pre-disturbance data on growth and only the damage surveys and distribution of trees after the fourth growing season following the storm. However, we have data on the fate of every tree that existed at that time and all that have grown to DBH > 2.5 cm during the next 22 years.

Growth rate following the hurricane was calculated using linear regression of total BA vs. year. The slope of the regression line provides the best estimate of average BA growth of that species. The value and significance of the regression coefficient (r) also determine the direction and linearity of the relationship. Using the sum of tree and sapling BA avoided variation associated with transition from sapling to tree. Finally, growth rates of selected species were compared using the “t” statistic to compare regression slopes.

## 3. Results

The first ten years of results [14] included upland hardwood and longleaf pine types at Hobcaw that were lost to management activities; thus, this paper only includes data from the 42 remaining plots (Table 1). Simple enumeration of the number and BA of each species, in each timber type, and at each site, comparable to the original study [14], are contained in the text and eleven figures in the Supplementary Data. A ranked list of all species measured at each type and site are also contained in the Supplemental Data (Tables S1–S11).

Analysis to investigate the behavior of these four sites began with determining a subset of species that represented each site. These subsets were chosen for each site by examining the 2016 list of IVs to pick the top species that combined to account for over 80% of the site importance values. For each species and site, average growth for the entire 23-year period was determined by a linear regression of total BA of that species versus year. In the following figures, each vegetation type is represented by an initial BA graph and a growth rate graph. The initial BA data, with the initial damage survey [1,25,26,29], can be viewed to represent the x axis of the Bellingham et al. [18] analysis, resistance to disturbance. The growth rate data can be viewed as the y-axis in that same analysis.

### 3.1. Overall Site Differences

**Figure 3:**
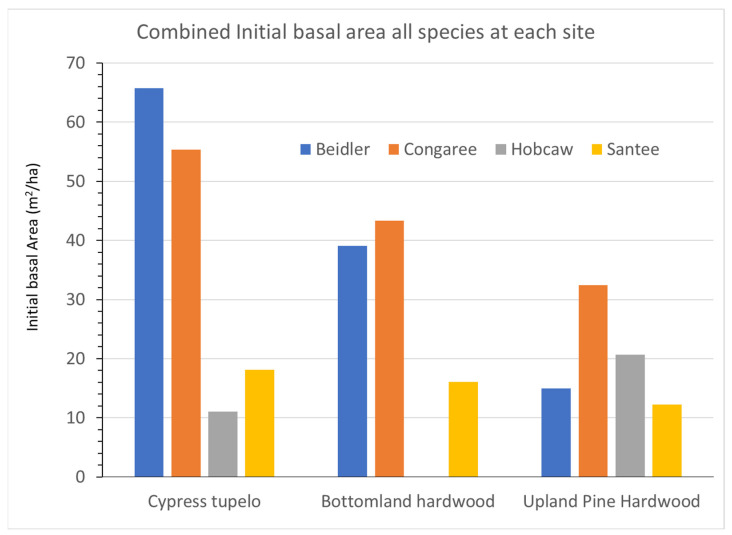
Initial basal areas of all species at each site for each vegetation type.

**Figure 4:**
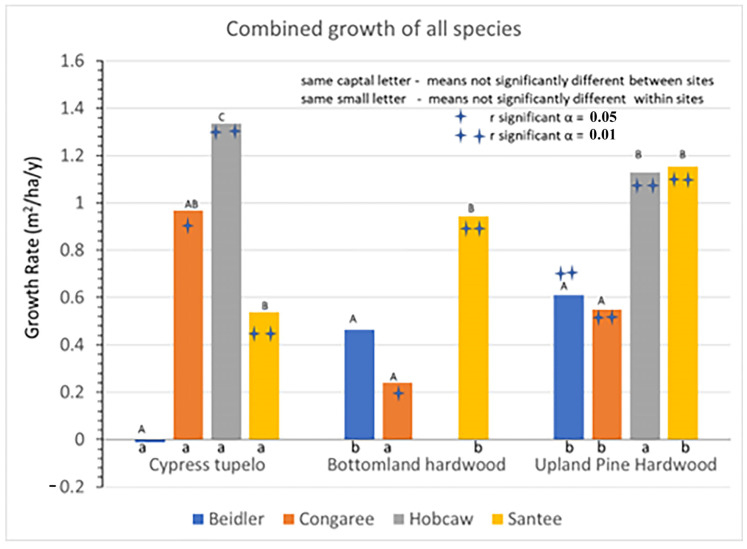
Overall growth rates of all species at each site for each vegetation type.

The four sites differed in several environmental factors and the level of hurricane damage. These differences contributed to differing initial BA (Figure 3) and growth rates (Figure 4) between the sites. Initial BA most likely reflect the degree of hurricane damage as they are consistent with the wind and saltwater tolerance of various species [30] and the likely wind velocities at each site. The cypress-tupelo vegetation type contains many wind-resistant species and showed very little damage at both the Beidler and Congaree sites; yet, at Santee, higher wind velocities resulted in much lower BA even four growing seasons after the storm. At Hobcaw the cypress-tupelo type was exposed to saline soil water until the summer of 1991 [28]. This site type had the lowest initial BA of all sites. The bottomland hardwood type showed similar relationship between the sites but had overall lower initial BA. That would also be consistent with resistance to wind damage as the type has both intermediate and low resistance species in this type. The upland pine hardwood type, including the ridge bottom type at Beidler, had the least wind resistant species and showed the lowest initial BA of all sites. The relationship of initial basal area BA to vegetation type was evident at both Beidler and Congaree, but was doubtful at Santee. At Hobcaw any relationship to wind resistance was overshadowed by the strong mortality due to saltwater.

The major strength of this study was the ability to track individual populations of trees over a 23-year period, allowing quite precise estimates of growth at the individual, site, and vegetation type levels (Figure 4). Higher precision in measuring growth aides in the interpretation of the overall site growth rates. At both Beidler and Congaree, the initial BA was over 50 m^2^/ha. The growth at Beidler was near 0 and insignificant, while the growth at Congaree was nearly 1 m^2^/ha/y, and significantly different from zero, yet the two site growth rates were not significantly different. Both types contained species with significant growth and others with significant decline resulting in a poorer estimate of the growth rates at each site and very poor discrimination between the sites. Likewise, the poorer estimates of growth do not allow discrimination between the cypress-tupelo and bottomland hardwood type. However, estimates of subsequent growth at the Hobcaw and Santee sites, where damage was much higher, are highly significant and smaller differences between types are more easily discriminated. These data suggest that light availability was much more important for recovery than any differences in wetland characteristics. The behavior of individual species may provide more insight into hurricane recovery of these vegetation types.

### 3.2. Cypress-Tupelo Vegetation Type

**Figure 5:**
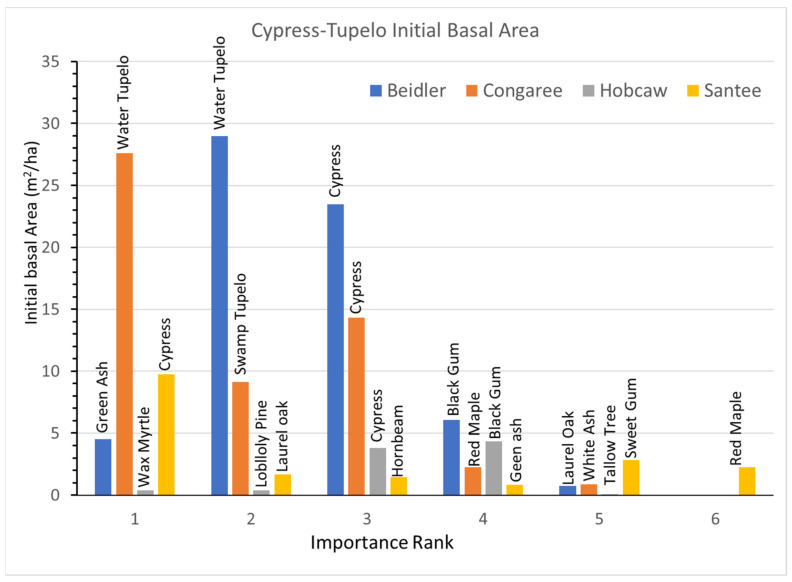
Distribution of initial basal area of each species, by importance rank (1 means the most important, while 6 means the least important) for each study site of the cypress-tupelo vegetation type.

**Figure 6:**
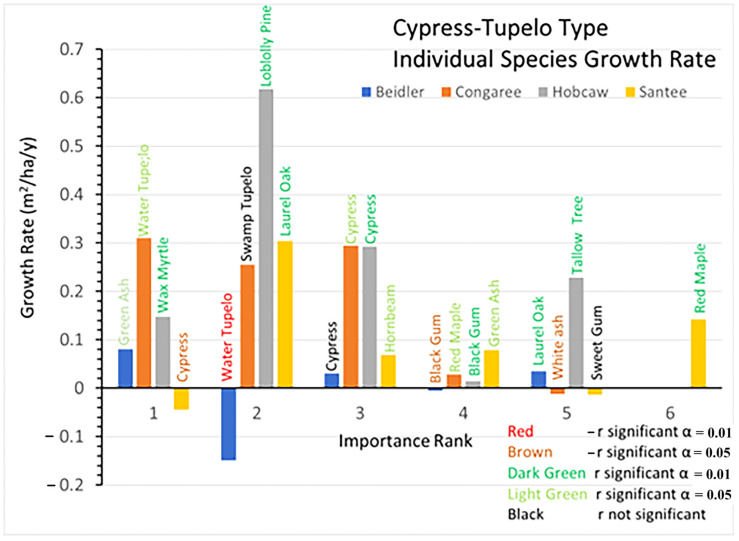
Distribution of basal area growth of species, by importance factor (1 means the most important, while 6 means the least important), for each study site of the cypress-tupelo vegetation type.

Initial BA and BA growth rates for the dominant species are presented in Figure 5 (initial BA) and Figure 6 (total BA growth). These figures, and the four following for the other vegetation types, portray several relationships. The bar height is the primary variable (initial BA or growth), and the x axis is the importance rank of each species. The bars are color coded for each site, the individual species names are listed above each bar, and on the BA growth graphs the sign and significance of the growth rate equation (r) is portrayed by the color of the species name. Note also that the y scale changes with the magnitude of the largest reading

The initial BA (Figure 3) indicated that there were only a few small changes from the damage assessments made after the storm. Cypress and water tupelo continued to dominate the Beidler and Congaree sites where damage to these species was slight [25,29]. At Santee cypress dominated the sparse forest there in 1993, and at Hobcaw the few remaining cypress and black gum (Supplementary Data Figure S7A) also represented the greatest BA.

Growth rates since 1993 show great differences among the sites in both overall growth, but also by a variety of individual species differences. At Beidler water tupelo showed a highly significant decrease in BA and black gum showed a significant decline (Figure 6). Only green ash and laurel oak showed significant growth. Although the growth rate of cypress nearly equaled laurel oak, cypress growth was not significantly different from 0. Cypress showed a decline in the number of trees after 2007 (Supplementary Data Figure S1B). At Congaree cypress, water tupelo, and swamp tupelo continued to dominate the stand. Swamp tupelo growth was not significant at Congaree, and there was also a decline in the number of trees after 2007, which corresponded to a sharp increase in the number of red maple saplings (Supplementary Data Figure S4B). At Hobcaw the surviving cypress grew quite well while the black gum had significant, but very slow growth. However, the invading tallow tree and native wax myrtle grew at about the same pace as cypress, while the loblolly pine grew nearly twice as fast. At Santee BA of cypress declined significantly during the period, while the laurel oak and red maple both had highly significant greater growth. The hornbeam and green ash also showed significant but moderate growth at Santee.

### 3.3. Bottomland Hardwood Vegetation Type

Bottomland hardwoods had a greater number of species representing 80% of the IV, seven as opposed to the five and six in the cypress-tupelo type. The bottomland hardwood type was more widely damaged at Beidler and Santee than the cypress tupelo type [25,29] and this type was not studied at Hobcaw. At Santee the bottomland hardwood type was slightly up slope from the cypress tupelo stands [14].

**Figure 7:**
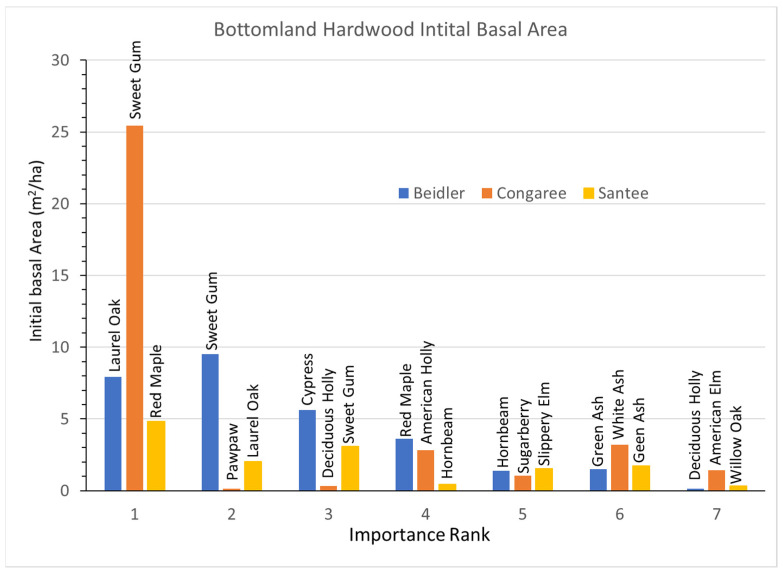
Distribution of initial basal area of species, by importance factor (1 means the most important, while 7 means the least important), for each study site of the bottomland hardwood vegetation type.

In the Beidler Forest the bottomland hardwood type was intermediate in the amount of damage between the low damage cypress tupelo type and the high damage ridge bottom type [29]. The initial BA graphs indicates that relative BA and IVs are well matched (Figure 7). Unlike some highly disturbed sites, no species was represented by a large number of small trees, and BA was quite evenly distributed in laurel oak, sweetgum, and cypress. Other than cypress, all the species in this type were also intermediate in their resistance to wind damage [30]. The Congaree site was unique as that site was nearly completely dominated by sweetgum with small contributions by other species. Pawpaw had a high IV based on numerous small trees (Supplementary Data Figure S5B). At Santee the BA of species were more evenly divided among the seven most important species, although all the values were relatively small.

**Figure 8:**
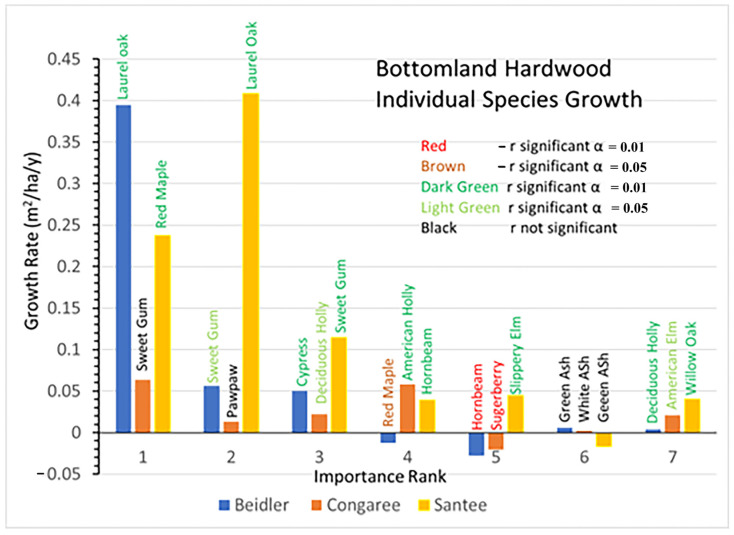
Distribution of basal area growth of species, by importance factor (1 means the most important, while 7 means the least important), for each study site of the bottomland hardwood vegetation type.

Growth rates also highlight the very different reactions of the Congaree site to the other two (Figure 8). At Congaree the dominant sweetgum grew quite slowly as did most of the other species. Only the two hollies and American elm had significant positive growth, while sugarberry declined significantly. At Beidler laurel oak growth was greater than the sum of the next six most important species combined, and there was significant negative growth for red maple and hornbeam. At Santee laurel oak also had the largest growth rate. However, at Santee five of the other six important species also showed significant positive growth.

### 3.4. Upland Pine Hardwood Type

**Figure 9:**
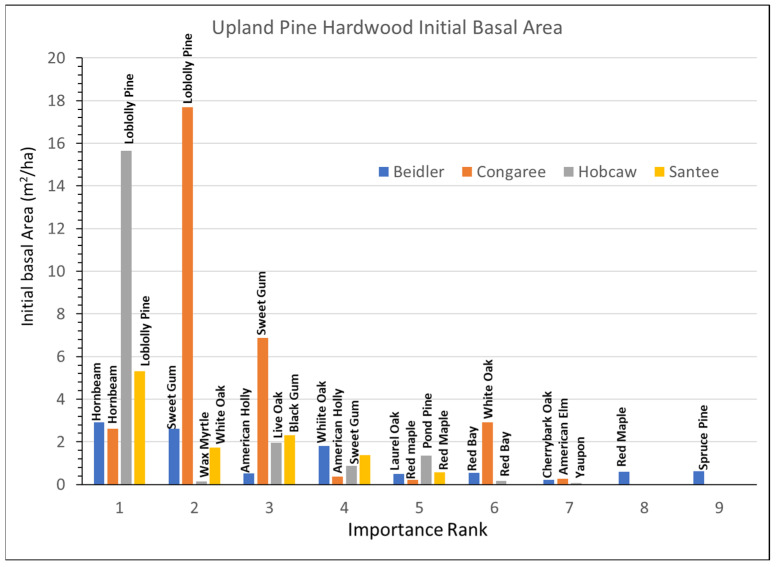
Distribution of initial basal area of species, by importance factor (1 means the most important, while 9 means the least important), for each study site of the upland pine-hardwood vegetation type.

The upland pine hardwood type was represented by the slightly different ridge bottom sites at Beidler. The important species had some similarities to the other the sites (Figure 9), but the pine species there was spruce pine instead of loblolly, the pine species at the other three sites. At Beidler spruce pine was especially susceptible to wind damage with over 90% mortality [29]. At the other three sites, loblolly pine represented an important part of the type. The Congaree site was only represented by a single 0.2 ha plot and was located near the edge of the floodplain [14], and was not part of the damage assessment [29] which found high mortality of very large loblolly found in the bottomland hardwood type. The 1994 upland pine hardwood type at Congaree had smaller trees and included many small hornbeam (Supplementary Data Figure S6B). The Hobcaw site also reflected lesser damage of loblolly pine associated with the lower wind speeds at Hobcaw. Although the upland pine hardwood site at Hobcaw was also covered by the tidal surge, it was less impacted by salination due to over 250 mm of rain in a subsequent tropical storm in October 1989 [27,28]. At Santee loblolly pine was also dominant, but wind damage lowered the BA of all species there.

**Figure 10:**
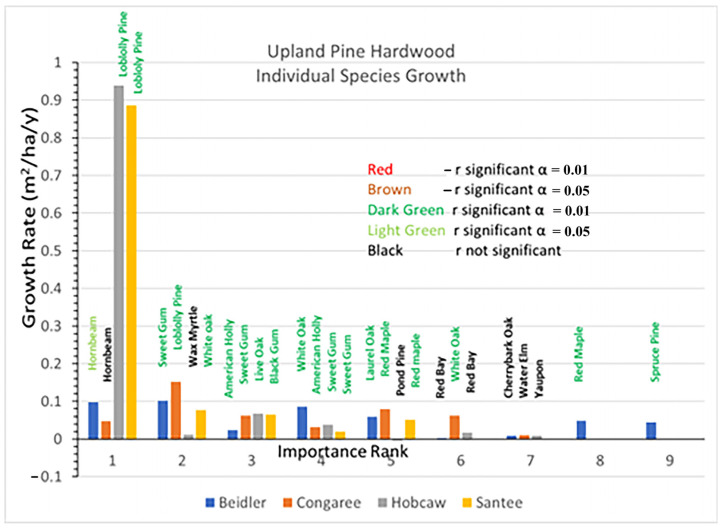
Distribution of basal area growth of species by importance factor (1 means the most important, while 9 means the least important) for each study site of the upland pine-hardwood vegetation type.

Rapid growth of loblolly pine has been evident at both the Hobcaw and Santee sites and dominated the response to the hurricane at those sites (Figure 10). Highly significant loblolly growth was also greatest at the Congaree site although, at 0.15 m^2^/ha/y, it was much slower than loblolly at either Hobcaw or Santee. Spruce pine growth at Beidler was highly significant but the few survivors made up a small portion of the species there. Hornbeam at Beidler and Congaree and sweetgum at Congaree grew much faster in the upland pine hardwood type than those same species in the bottomland hardwood type.

## 4. Discussion

The pre-disturbance composition and structure of the forest, the level of hurricane intensity in the area, and the past exposure of the forest to other disturbance events are important factors in determining the long-term effects of hurricane disturbance [16,26]. The four sites sampled for this study were all subjected to varying degrees of hurricane intensity. They also differ in soils, geographic setting, water source and resistance to hurricane damage of individual species at each site [30]. In Figure 3, there is clear evidence of wide differences in the initial BA of the four sites after the storm as measured in 1993. Both Beidler and Congaree had large BA in the cypress-tupelo type with less in the bottomland hardwoods, and even less in the upland pine hardwood types. These quantities are consistent with the species make up of these vegetation types (Figure 5, Figure 7 and Figure 9). At Beidler this was particularly evident with least susceptible species in the cypress tupelo site and most susceptible species in the ridge bottom type [29]. At these sites, regrowth has been variable with no significant growth at the Beidler cypress-tupelo and bottomland hardwood types and quite variable growth at Congaree. At Beidler many species had sufficient mortality to record negative growth for the measured 23-year period (Figure 6 and Figure 8).

At Santee and Hobcaw there was much less difference in the initial BA of the different types. Much higher wind speeds at Santee (Figure 2) resulted in loss of most of the larger trees regardless of resistance to wind damage [1], and all types had less than 20 m^2^/ha of BA in 1993. At Hobcaw both cypress-tupelo and upland hardwood type were subject to saltwater intrusion and high mortality. Damage was more severe in the cypress-tupelo type where subsequent groundwater flow enhanced mortality [28]. Regrowth of both types at Hobcaw were highly significant and the highest value of any site for the cypress-tupelo type, and higher than all except Santee for the pine hardwood type. Likewise, at Santee lower initial BA of bottomland hardwood and upland pine hardwood types were followed by growth that was significantly greater than the other sites and significantly greater than the cypress-tupelo site at Santee.

Factors other than wetland vegetation types appear to be much more important to the reaction to hurricane disturbance. The extent of damage caused by wind or salt water seemed to be the most determinative factor in vegetation recovery. The results seem to support the idea that individual surviving species self-organize into new stable associations. Bellingham et al. [18], examining montane forests in Jamaica following Hurricane Gilbert, found similar species level differences and concluded recovery can be best described by the concept described in Figure 2. That concept, like many others stressing a Gleasonian view of ecosystem development [12,13,14,17], was published well after this study was initiated, but the data collected in this study can be retrospectively analyzed to examine that concept as applied to temperate forests of the southeastern U.S.

**Figure 11:**
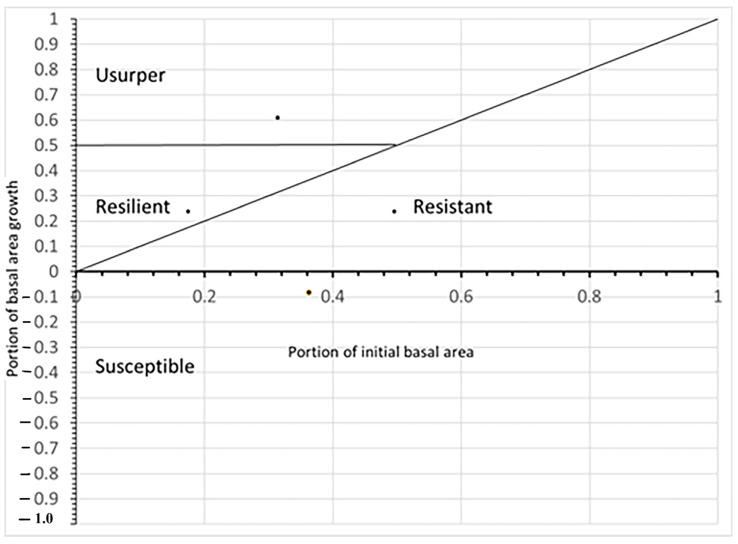
Revised version of the concept shown in Figure 1 modified to reflect the data collected in this study. In this analysis portion of the total growth represented by a species is compared to an initial portion of basal area rather than pre-disturbance growth.

Evaluation of the concept needed to be modified as previous uses of this concept [18,19] included measures of species growth prior to disturbance. The concept could be easily viewed as binary; species that were heavily damaged were either susceptible if they grew slower after the disturbance or resilient if they grew faster, and species that mostly survived the disturbance were either resistant if they grew the same or slower or usurpers if they grew faster. In this study, there was no pre-hurricane growth data, so the simple binary choices had to be modified (Figure 11). At each site the initial BA of a species was normalized by dividing by the total BA for that site and type; for example, at Hobcaw cypress initial BA on the cypress type was 3.8 m^2^/ha and the total BA of Hobcaw cypress-tupelo type was 11.0 m^2^/ha, so the normalized value as 0.34 or 34%. Likewise, the cypress growth rate at Hobcaw cypress-tupelo type was 0.29 m^2^/ha/y while the growth rate of the entire type was 1.34 m^2^/ha/y for a normalized value of 0.22 or 22%. Each species at each site and type similar portion of initial BA and portion of BA growth and were calculated and plotted onto graphs similar to Figure 11.

The rationale evaluating for the Bellingham concept in this way was: (1) Any species that had negative growth was susceptible; (2) Any species with less than 50% of the initial BA, but represented a higher proportional growth than its portion of initial BA, was resilient; (3) Any species with more than 50% portion of BA growth was usurper; and (4) Any species that represented a smaller portion of BA growth than its initial BA, but still showed positive growth, was considered resistant.

### 4.1. Species Responses in Cypress-Tupelo Type

**Figure 12:**
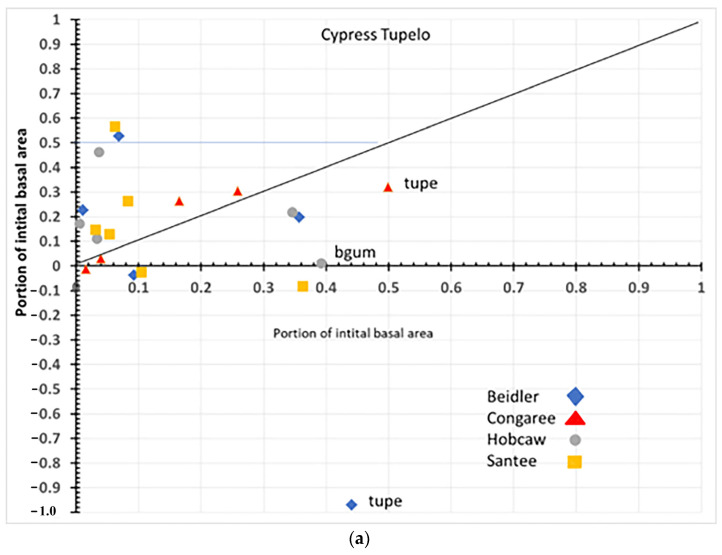

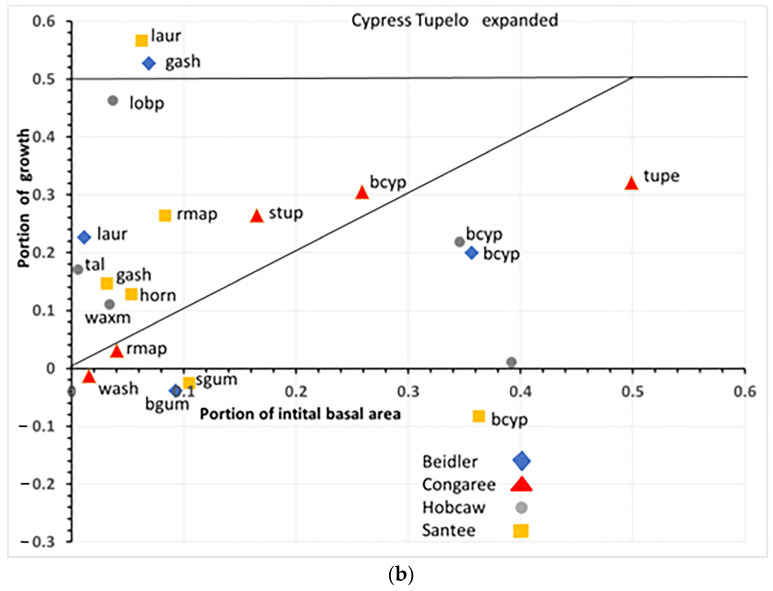
(**a**) Overall response of species in the cypress-tupelo type. Each species is labeled with four letter species code from Table 1 and symbols representing sites. (**b**) Enlargement of section of (**a**) (cypress-tupelo) near the origin in order to separate species codes.

Responses of species are not uniform on the four sites, nor do they reflect the initial vegetation type in all cases (Figure 12a,b). Only at Congaree are the three defining species cypress, water tupelo, and swamp tupelo resistant and resilient suggesting the type at Congaree is quite stable. At Beidler both water tupelo and blackgum are susceptible. Water tupelo at Beidler not only is susceptible but it accounts for nearly 90% of the negative growth at the site. Water tupelo growth there was not only negative, but highly significant and had negative linear growth for the 23-year period (Figure 6). At Beidler water tupelo had such large mortality the net growth of all species was near zero. To evaluate the role of trees with positive growth, the sum of all positively growing species was used as total plot growth in analysis of the role of those species. At Santee, there were no surviving tupelo, and cypress was a susceptible species. This site type was being usurped by laurel oak that was accountable for nearly 60% of the growth at the site. At Hobcaw cypress was resistant, very similar to Beidler, but growth was predominantly loblolly pine with the invasive tallow tree having BA growth not significantly different from cypress (probability of greater t = 0.083).

### 4.2. Species Responses in the Bottomland Hardwood Type

**Figure 13:**
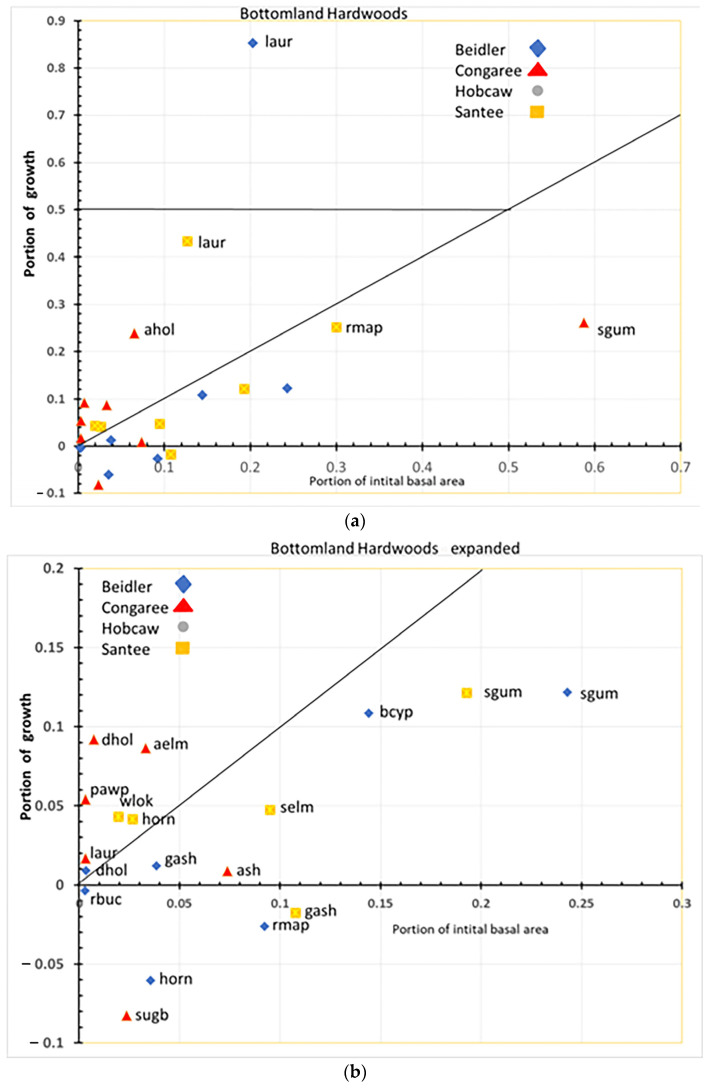
(**a**) Overall response of species in the bottomland hardwood type. Each species is labeled with four letter species code (Table 1) and symbol for site. (**b**) Enlargement of section of (**a**) (bottomland hardwood) near the origin in order to separate species codes.

The bottomland hardwood type also showed a remarkable difference in the response of species at each site (Figure 13a,b). Laurel oak performed an important role in regrowth at both Beidler and Santee. At Beidler, it was a very strong usurper with over 84% of the total growth in the type there. At Santee, laurel oak also grew better than any other species as highly resilient. At Santee, red maple was the second most rapidly growing but still had a lower portion of growth than of initial BA, making it a resistant species there. Initially, red maple was represented by a high number of small trees and numerous saplings (Supplementary Data Figure S10A,B) suggesting its resistance was more as advanced reproduction than as large trees. At Congaree, sweetgum dominated the site in initial BA and showed modest growth and was solidly classed as resistant. As with the cypress-tupelo type, bottomland hardwoods at Congaree changed relatively little over the period. American holly and many species that were lesser components of the initial BA showed high resiliency (Figure 13b). At Congaree, laurel oak was also resilient but was always a minor component of the type.

The bottomland hardwood type had a larger number of susceptible species than the other types. Green ash at Santee, sugarberry at Congaree, and red maple, hornbeam, and red buckeye at Beidler all showed greater mortality than growth. At Santee, both red maple and hornbeam were resistant and resilient, respectively. It appears that strong growth of laurel oak at Beidler was leading to decline of these species. The difference between Beidler and Santee relates to the relative size of red maple and laurel oak at those sites. As previously stated, red maple at Santee were primarily advanced reproduction and poised for rapid growth and grew nearly as quickly as laurel oak. However, at Beidler the initial sizes of red maple and laurel oak reversed, with laurel oak being represented by small trees and many saplings and red maple as fewer, larger trees and relatively fewer saplings (Supplementary Data Figure S2A,B). The behavior of these two species at these sites implies a complex relationship of wind damage, initial diameter distribution, and the inherent competitiveness of the species involved.

### 4.3. Species Response in the Upland Pine Hardwood Type

**Figure 14:**
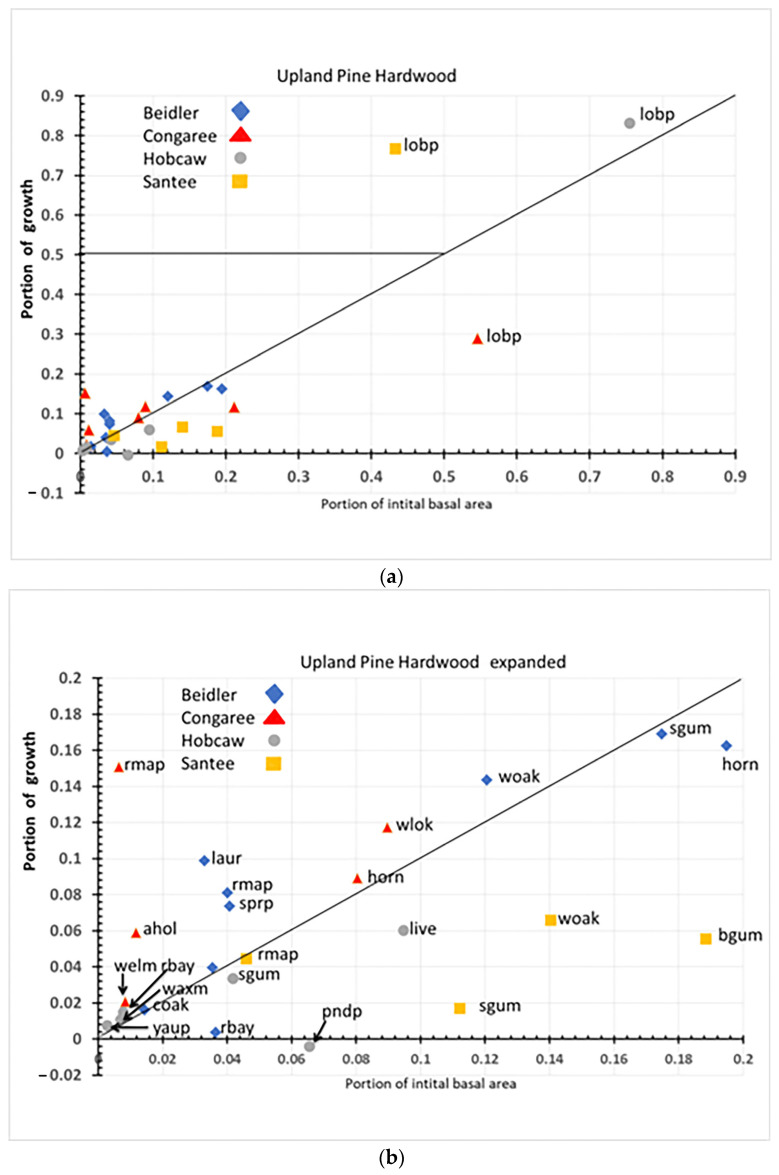
(**a**) Overall response of species in the upland pine hardwood type. Each species is labeled with 4 letter species code (Table 1) and site name. (**b**) Enlargement of section of (**a**) near the origin in order to separate species codes.

Loblolly pine was, by far, the most important species of this type on three of the four sites (Figure 14a). At the two most damaged sites, Hobcaw and Santee it filled the role of usurper even though it represented 75% of the initial basal area at Hobcaw. If we disregard the large impact of loblolly pine (Figure 14b), we can see that there are similar species, and they act similarly in both the bottomland hardwood and pine hardwood types.

Sweetgum is resistant at all upland pine hard wood sites as it was on all three bottomland hardwood sites. American holly is resilient at Congaree and Beidler in the upland pine hardwood type as it was in the Congaree bottomland hardwoods. Water, willow, and laurel oaks were at least resilient wherever they occurred. In contrast to the species that were similar, red maple was present at some sites in all three vegetation types, yet its role varied widely among sites. In the cypress tupelo type, it was resilient at Santee and resistant at Congaree. In the bottomland hardwoods, it was again resilient at Santee but susceptible at Beidler. In the pine hardwoods, it was resistant at Santee and resilient at Congaree. Finally, the role of soil salinization at Hobcaw can be seen in the role of the invasive Chinese tallow and the shrubby wax myrtle and red bay; all occurring as resilient species, in both the cypress tupelo type and the upland pine hardwood type.

### 4.4. General Reactions

Usually, pre-hurricane composition of a disturbed forest is a determinant of the impacts that the disturbance may have on forest structure and composition [25,26,29]. In this study, which was the case only for sites at Congaree and the cypress-tupelo type at Beidler, the cypress and tupelo species were resistant and able to continue to dominate at the Beidler site with wind damage from speeds of up to 50 m/s. The dominant sweet gum in the Congaree bottomland hardwood type was resistant at 45–50 m/s, as did the smaller loblolly pines in the upland pine hardwood type. All the other sites showed profound changes in vegetation in the recovering stands.

The cypress-tupelo type showed the most profound change in vegetation where wind and saltwater impacts were the greatest. At Santee laurel, oak has become a usurper species and red maple highly resilient, while cypress has shown significant negative growth. At Hobcaw loblolly pine, shrubby wax myrtle, and non-native Chinese tallow made up 74% of all growth in the cypress tupelo site. Cypress made up most of the other 25% with blackgum being nearly stagnant there for 23 years. Even at Beidler, where the cypress and water tupelo made up most of the initial basal area, green ash was growing faster than everything else. Much of the reason for the success of green ash may have been because water tupelo had the most negative growth (highest mortality) of any species on any site.

In the bottomland hardwood type, there were profound changes in the species that have grown best during the period, at all sites other than Congaree. At Beidler laurel oak accounted for 84% of all growth on the site. The greatest number of susceptible species occurred in the bottomland hardwoods at Beidler. Red maple, hornbeam, and red buckeye all showed negative growth there. Beidler was the only site where hornbeam growth was negative, as it was usually resilient at most sites where it occurred. At Santee, laurel oak and red maple also made up 68% of all growth on the site. Green ash showed negative growth but that was not statistically significant.

In the upland pine hardwood type loblolly pine was a usurper at both Hobcaw and Santee. At these sites, the pine hardwood type was progressing nearer to pure pine stands. These two sites also had significantly greater overall growth than Congaree and Beidler. The lack of loblolly pine a Beidler limits the insight that site can provide for this type. The name ridge bottom was certainly more appropriate as it had more in common with the bottomland hardwood type than with the other upland pine hardwood sites.

As noted previously, the rate of growth at each site was inversely proportional to the initial BA. The rates of greatest growth were also at sites where loblolly pine was either very resilient or usurper. Rapid growth of loblolly pine in early succession has been well established [31,32]. The growth of, primarily, loblolly pine led to the highest growth rate at the Hobcaw cypress-tupelo site. Soil salinization may have performed a part in this rapid growth as competition control by herbicides is used to improve commercial loblolly pine plantations [33]. The presence of wax myrtle, a known nitrogen fixer [34], may also have contributed to the superior growth at this site.

One more subtle aspect is also evident when species were classified with the Bellingham [18] concept of response. On three of the cypress-tupelo sites, obligate wetland species (cypress and tupelo, [24]) were being replaced by facultative wet (green ash, or laurel oak), or merely facultative (loblolly pine) as usurper or highly resilient. Likewise, on the bottomland hardwood sites many of the susceptible species (green ash, red maple, sugarberry) were facultative wet while resilient species (American holly, or hornbeam) were facultative. Although it was less clear in the bottomland hardwood type there has been a replacement of obligate and facultative wetland species with species that are also common on uplands. At Santee, air temperature has risen 0.19 °C per decade while rainfall has not changed significantly [35]. Rising temperature could increase evaporative demand and may have resulted in the sites becoming drier over the past two decades.

## 5. Conclusions

We have applied the recovery concepts of Bellingham et al. [18] to species common to temperate forests of the southeastern US, by examining recovery trends of three forest types from four sites for 23 years, from years 4–27 after Hurricane Hugo. Similar classifications have been applied to tropical forest species [6,36,37]. These classifications are broad generalizations and are often specific to a stand or a population. Here, we found species may be assigned to more than one role based on initial damage. This was most important for wind resistant species, as estimated wind speeds varied form 35–60 m/s on the various sites studied. The highly resistant cypress and tupelo became susceptible on sites with highest wind speeds. Loblolly pine and laurel oak were consistently very resilient or usurper on all sites where the original forest was destroyed by wind or saltwater. In contrast, red maple varied from highly resilient to resistant to susceptible, depending on the site and type where it occurred. One unique aspect of this classification was a clear indication that the species of the wetland sites are shifting from obligate wetland species to species that are only common to wetlands (facultative wet) or even species that grow equally well on upland and wetlands (facultative).

The results of this study suggest that orderly succession does not explain the behavior of southeastern coastal forests to hurricane disturbance. Instead, these forests appear to have self-organized into new ecosystem organizations following disturbance. Self-organization of species into ecosystems has become a leading idea of ecological science [38,39], leading to new understanding of ecosystem stability [40]. The concept has even been expanded beyond ecology into industrial control systems [41].

After Hurricane Hugo the position of the stand in relation to the wind field was the most important factor in determining how the forest reorganized. One unique realization occurred on the cypress tupelo site at Hobcaw. Soil salinization and release of a nitrogen fixing species have resulted in a natural stand that approximates intensive pine plantation forestry. However, those conditions also resulted in the invasive Chinese tallow to thrive in the stand as it has done on bottomland hardwood forests of south-central Louisiana [38].

The intensity of hurricanes may increase in the future as sea surface temperatures rise with climate change [42,43,44,45], Such an increase will likely result in more stands being exposed to strong wind fields and saltwater intrusion near the coast. These results suggest that species that survive primarily by resistance will be more likely to be replaced by resilient species, such as the laurel oak and loblolly pine did on the sites studied here.

## Figures and Tables

**Table 1:** A complete list of graphics code, common name, and taxonomic name for all species in this study.

Graphic Code	Common Name	Taxonomic Name
adog	dogwood	*Cornus* spp.
aelm	American elm	*Ulmus americana* L.
ahol	American holly	*Ilex opaca* Aiton
ash	ash	*Fraxinus* spp.
bacc	baccharis	*Baccharis halimifolia* L.
bcyp	bald cypress, cypress	*Taxodium distichum* (L.) Rich.
bgum	blackgum	*Nyssa sylvatica* Marsh.
bech	beech	*Fagus grandifolia* Ehrh.
bitr	bitternut hickory	*Carya cordiformis* (Wangenh.) K. Koch
bjok	blackjack oak	*Quercus marilandica* Münchh.
blko	black oak	*Quercus velutina* Lam.
blue	blueberry	*Vaccinium elliottii* Chapm.
boxe	boxelder	*Acer negundo* L.
butn	buttonbush	*Cephalanthus occidentalis* L.
cash	Carolina ash	*Fraxinus caroliniana* Mill.
cbok	cherrybark oak	*Quercus pagoda* Raf.
cedr	eastern redcedar	*Juniperus virginiana* L.
cher	black cherry	*Prunus serotina* Ehrh.
coak	swamp chestnut oak	*Quercus michauxii* Nutt.
cwil	coastal plains willow	*Salix caroliniana* Michx.
dahn	dahoon holly	*Ilex cassine* L.
dhol	deciduous holly	*Ilex decidua* Walter
dogw	flowering dogwood	*Cornus florida* L.
elm	elm	*Ulmus* spp.
gash	green ash	*Fraxinus pennsylvanica* Marsh.
hawt	hawthorn	*Crataegus* spp.
hick	hickory	*Carya* spp.
horn	hornbeam, ironwood	*Carpinus caroliniana* Walter
hsug	horse sugar	*Symplocos tinctoria* (L.) L’Hér.
inkb	inkberry	*Ilex glabra* (L.) A. Gray
laur	laurel oak	*Quercus laurifolia* Michx.
live	live oak	*Quercus virginiana* Mill.
lobp	loblolly pine	*Pinus taeda* L.
long	longleaf pine	*Pinus palustris* Mill.
mhik	mockernut hickory	*Carya tomentosa* (Lam.) Nutt.
mulb	red mulberry	*Morus rubra* L.
ocup	overcup oak	*Quercus lyrata* Walter
pers	persimmon	*Diospyros virginiana* L.
pawp	pawpaw	*Asimina triloba* (L.) Dunal
phik	pignut hickory	*Carya glabra* (Mill.) Sweet
plan	water elm	*Planera aquatica* J.F. Gmel.
pndp	pond pine	*Pinus serotina* Michx.
poak	post oak	*Quercus stellata* Wangenh.
rbay	redbay	*Persea borbonia* (L.) Spreng
rbuc	red buckeye	*Aesculus pavia* L.
rhik	shagbark hickory	*Carya ovata* (Mill.) K. Koch
rmap	red maple	*Acer rubrum* L.
sbay	sweetbay	*Magnolia virginiana* L.
sdog	swamp dogwood	*Cornus foemina* Mill.
selm	slippery elm	*Ulmus rubra* Muhl.
sgum	sweetgum	*Liquidambar styraciflua* L.
shok	Shumard oak	*Quercus shumardii* Buckley
shrt	shortleaf pine	*Pinus echinata* Mill.
spic	spicebush	*Lindera benzoin* (L.) Blume
sprp	spruce pine	*Pinus glabra* Walter
sred	southern red oak	*Quercus falcata* Michx.
stor	snowbell	*Styrax* spp.
stup	swamp tupelo	*Nyssa biflora* Walter
sugb	sugarberry	*Celtis laevigata* Willd.
sycm	sycamore	*Platanus occidentalis* L.
tall	Chinese tallow	*Triadica sebifera* (L.) Small
tupe	water tupelo	*Nyssa aquatica* L.
vibo	viburnum	*Viburnum* spp.
vwil	Virginia willow	*Itea virginica* L.
wash	white ash	*Fraxinus americana* L.
waxm	wax myrtle	*Morella cerifera* (L.) Small
welm	winged elm	*Ulmus alata* Michx.
whik	water hickory	*Carya aquatica* (Michx. f.) Nutt.
whol	winterberry	*Ilex verticillata* (L.) A. Gray
will	black willow	*Salix nigra* Marsh.
wloc	water locust	*Gleditsia aquatica* Marsh.
wlok	willow oak	*Quercus phellos* L.
woak	water oak	*Quercus nigra* L.
wsum	winged sumac	*Rhus copallinum* L.
yaup	yaupon	*Ilex vomitoria* Aiton
ypop	yellow poplar	*Liriodendron tulipifera* L.

**Table 2:** Number of study plots present among four major sites and five forest cover types.

Cover Type	Beidler Forest	Congaree Park	Santee Forest	Hobcaw	TOTAL
Cypress Swamp	4	4	4	4	16
Bottomland Hardwood	4	5	4		13
Upland Pine-Hardwood		1	4	4	9
Ridge Bottom	4				4
**TOTAL**	12	10	12	8	42

## Data Availability

The raw data supporting the conclusion of this article will be made available by the authors, without undue reservation.

## Supplementary Materials

The following supporting information can be downloaded at: https://www.mdpi.com/article/10.3390/plants12040691/s1, Figure S1: (A) Basal area and (B) density changes for dominant species in the cypress-tupelo type at Beidler Forest; Figure S2: (A) Basal area and (B) density changes for dominant species in the bottomland hardwood forest type at Beidler Forest; Figure S3: (A) Basal area and (B) density changes for dominant species in the ridge bottom forest type at Beidler Forest; Figure S4: (A) Basal area and (B) density changes for important species in the cypress-tupelo forest type at Congaree National Park; Figure S5: (A) Basal area and (B) density changes for important species in the bottomland hardwood forest type at Congaree National Park; Figure S6: (A) Basal area and (B) density changes for important species in the pine hardwood forest type at Congaree National Park; Figure S7: (A) Basal area and (B) density changes for important species in the cypress-tupelo forest type at Hobcaw Barony; Figure S8: (A) Basal area and (B) density changes for important species in the upland pine-hardwood forest type at Hobcaw Barony; Figure S9: (A) Basal area and (B) density changes for important species in the cypress-tupelo forest type at Santee Experimental Forest; Figure S10: (A) Basal area and (B) density changes for important species in the bottomland hardwood forest type at Santee Experimental Forest; Figure S11: (A) Basal area and (B) density changes for important species in the upland pine-hardwood forest type at Santee Experimental Forest; Tables S1–S11: ID code, scientific name, and Importance Value (IV) for tree species.
