# Supplementary material for: Twenty-Seven Year Response of South Carolina Coastal Plain Forests Affected by Hurricane Hugo
Source: Plants (Basel). 2023 Feb 4;12(4):691. doi: 10.3390/plants12040691 (PMC9967406; doi:10.3390/plants12040691)
Supplement: Supplementary file 1 [file plants-12-00691-s001.zip › plants-2168833-supplementary.pdf]

Supplemental Data

Importance values in 2016 for all sites, Tables S1-S11

Table S1. ID code, scientific name, and Importance Value (IV) for tree species in Beidler Forest cypress-tupelo vegetation type.

Species code	Common Name	Scientific Name	IV(%)
gash	green ash	*Fraxinus pennsylvanica* Marsh.	36.652
tupe	tupelo	*Nyssa spp.*	27.939
bcyp	baldcypress	*Taxodium distichum* (L.) Rich.	21.923
bgum	black gum	*Nyssa sylvatica* Marsh.	6.256
laur	laurel oak	*Quercus laurifolia* Michx.	2.803
rmap	red maple	*Acer rubrum* L.	1.216
sdog	swamp dogwood	*Cornus foemina* Mill.	0.635
wloc	water locust	*Gleditsia aquatica* Marsh.	0.584
selm	slippery elm	*Ulmus rubra* Muhl.	0.465
aelm	American elm	*Ulmus americana* L.	0.420
sgum	sweetgum	*Liquidambar styraciflua* L.	0.355
ocup	overcup oak	*Quercus lyrata* Walter	0.204
elm	elm	*Ulmus* spp.	0.097
ash	ash	*Fraxinus* spp.	0.088
will	black willow	*Salix nigra* Marsh.	0.069
wlok	willow oak	*Quercus phellos* L.	0.048
wash	white ash	*Fraxinus americana* L.	0.046
butn	buttonbush	*Cephalanthus occidentalis* L.	0.045
vwil	Virginia willow	*Itea virginica* L.	0.044
unkn	unknown	unknown	0.022
boxe	boxelder	*Acer negundo* L.	0.022
adog	dogwood	*Cornus* spp.	0.022
ahol	American holly	*Ilex opaca* Aiton	0.022
TOTAL IV			99.978

Table S2. ID code, scientific name, and Importance Value (IV) for tree species in Beidler Forest bottomland hardwood vegetation type.

Species code	Common Name	Scientific Name	IV(%)
laur	laurel oak	*Quercus laurifolia* Michx.	28.464
sgum	sweetgum	*Liquidambar styraciflua* L.	13.258
bcyp	baldcypress	*Taxodium distichum* (L.) Rich.	8.762
rmap	red maple	*Acer rubrum* L.	8.509
horn	ironwood	*Carpinus caroliniana* Walter	8.133
gash	green ash	*Fraxinus pennsylvanica* Marsh.	6.924
dhol	deciduous holly	*Ilex decidua* Walter	4.514
rbuc	red buckeye	*Aesculus pavia* L.	2.480
tupe	water tupelo	*Nyssa aquatica* L.	2.411
elm	elm	*Ulmus* spp.	2.354
bgum	black gum	*Nyssa sylvatica* Marsh.	2.265
sdog	swamp dogwood	*Cornus foemina* Mill.	1.902
ocup	overcup oak	*Quercus lyrata* Walter	1.843
ahol	American holly	*Ilex opaca* Aiton	1.663
ash	ash	*Fraxinus* spp.	1.559
will	black willow	*Salix nigra* Marsh.	1.019
selm	slippery elm	*Ulmus rubra* Muhl.	0.082
cbok	cherrybark oak	*Quercus pagoda* Raf.	0.663
pers	persimmon	*Diospyros virginiana* L.	0.387
whik	water hickory	*Carya aquatica* (Michx. f.) Nutt.	0.384
aelm	American elm	*Ulmus americana* L.	0.329
hawt	hawthorn	*Crataegus* spp.	0.262
rbay	redbay	*Persea borbonia* (L.) Spreng.	0.253
welm	winged elm	*Ulmus alata* Michx.	0.189
butn	buttonbush	*Cephalanthus occidentalis* L.	0.162
woak	water oak	*Quercus nigra* L.	0.153
wloc	water locust	*Gleditsia aquatica* Marsh.	0.109
inkb	inkberry	*Ilex glabra* (L.) A. Gray	0.065
vibo	viburnum	*Viburnum* spp.	0.065

sugb	sugarberry	*Celtis laevigata* Willd.	0.033
bacc	baccharis	*Baccharis halimifolia* L.	0.032
TOTAL IV			100.000

Table S3. ID code, scientific name, and Importance Value (IV) for tree species in Beidler Forest ridge bottom vegetation type.

Species code	Common Name	Scientific Name	IV(%)
horn	hornbeam,ironwood	*Carpinus caroliniana* Walter	29.484
sgum	sweetgum	*Liquidambar styraciflua* L.	13.222
ahol	American holly	*Ilex opaca* Aiton	7.571
woak	water oak	*Quercus nigra* L.	7.432
laur	laurel oak	*Quercus laurifolia* Michx.	5.536
rbay	redbay	*Persea borbonia* (L.) Spreng	5.247
coak	swamp chestnut oak	*Quercus michauxii* Nutt.	4.697
rmap	red maple	*Acer rubrum* L.	4.463
sprp	spruce pine	*Pinus glabra* Walter	4.006
phik	pignut hickory	*Carya glabra* (Mill.) Sweet	2.218
rhik	shagbark hickory	*Carya ovata* (Mill.) K. Koch	1.883
elm	elm	*Ulmus* spp.	1.603
gash	green ash	*Fraxinus pennsylvanica* Marsh.	1.492
welm	winged elm	*Ulmus alata* Michx.	1.408
selm	slippery elm	*Ulmus rubra* Muhl.	1.178
bgum	black gum	*Nyssa sylvatica* Marsh.	1.092
lobp	loblolly pine	*Pinus taeda* L.	1.088
sdog	swamp dogwood	*Cornus foemina* Mill.	1.067
dhol	deciduous holly	*Ilex decidua* Walter	0.776
dogw	flowering dogwood	*Cornus florida* L.	0.571
ash	ash	*Fraxinus* spp.	0.563
aelm	American elm	*Ulmus americana* L.	0.504
cbok	cherrybark oak	*Quercus pagoda* Raf.	0.491
sbay	sweetbay	*Magnolia virginiana* L.	0.472
rbuc	red buckeye	*Aesculus pavia* L.	0.435
sugb	sugarberry	*Celtis laevigata* Willd.	0.359
blko	black oak	*Quercus velutina* Lam.	0.218
pers	persimmon	*Diospyros virginiana* L.	0.190
hawt	hawthorn	*Crataegus* spp.	0.167
ypop	yellow poplar	*Liriodendron tulipifera* L.	0.067

wlok	water locust	*Gleditsia aquatica* Marsh.	0.065
vibo	viburnum	*Viburnum* spp.	0.058
sugb	sugarberry	*Celtis laevigata* Willd.	0.055
unkn	unknown	unknown	0.039
mulb	red mulberry	*Morus rubra* L.	0.038
live	live oak	*Quercus virginiana* Mill.	0.031
bcyp	baldcypress	*Taxodium distichum* (L.) Rich.	0.027
mhik	mockernut hickory	*Carya tomentosa* (Lam.) Nutt.	0.027
hick	hickory	*Carya* spp.	0.022
ocup	overcup oak	*Quercus lyrata* Walter	0.021
tupe	water tupelo	*Nyssa aquatica* L.	0.020
cher	black cherry	*Prunus serotina* Ehrh.	0.020
waxm	wax myrtle	*Morella cerifera* (L.) Small	0.020
wsum	winged sumac	*Rhus copallinum* L.	0.019
adog	dogwood	*Cornus* spp.	0.019
shok	Shumard oak	*Quercus shumardii* Buckley	0.019
TOTAL IV			100.000

Table S4. ID code, scientific name, and Importance Value (IV) for tree species in Congaree cypress tupelo vegetation type.

Species code	Common Name	Scientific Name	IV(%)
tupe	water tupelo	*Nyssa aquatica* L.	32.946
stup	swamp tupelo	*Nyssa biflora* Walter	17.397
bcyp	baldcypress	*Taxodium distichum* (L.) Rich.	16.137
rmap	red maple	*Acer rubrum* L.	9.816
wash	white ash	*Fraxinus americana* L.	6.292
ahol	American holly	*Ilex opaca* Aiton	4.405
laur	laurel oak	*Quercus laurifolia* Michx.	2.929
sgum	sweetgum	*Liquidambar styraciflua* L.	2.819
plan	water elm	*Planera aquatica* J.F. Gmel.	1.329
horn	ironwood	*Carpinus caroliniana* Walter	1.108
sbay	sweetbay	*Magnolia virginiana* L.	0.910
rbay	redbay	*Persea borbonia* (L.) Spreng	0.813
dhol	deciduous holly	*Ilex decidua* Walter	0.566
ocup	overcup oak	*Quercus lyrata* Walter	0.385
sugb	sugarberry	*Celtis laevigata* Willd.	0.368
blue	blueberry	*Vaccinium elliottii* Chapm.	0.320
cash	Carolina ash	*Fraxinus caroliniana* Mill.	0.319
aelm	American elm	*Ulmus americana* L.	0.293
selm	slippery elm	*Ulmus rubra* Muhl.	0.123
whol	winterberry	*Ilex verticillata* (L.) A. Gray	0.120
welm	winged elm	*Ulmus alata* Michx.	0.099
cher	black cherry	*Prunus serotina* Ehrh.	0.088
bech	beech	*Fagus grandifolia* Ehrh.	0.081
woak	water oak	*Quercus nigra* L.	0.048
boxe	boxelder	*Acer negundo* L.	0.045
hawt	hawthorn	*Crataegus* spp.	0.045
cttn	eastern cottonwood	*Populus deltoides* W. Bart. ex Marsh.	0.042
gash	green ash	*Fraxinus pennsylvanica* Marsh.	0.040
butn	buttonbush	*Cephalanthus occidentalis* L.	0.040

unkn	unknown	unknown	0.020
hick	hickory	*Carya* spp.	0.040
TOTAL IV			100.000

Table S5. ID code, scientific name, and Importance Value (IV) for tree species in Congaree bottomland hardwood vegetation type.

Species code	Common Name	Scientific Name	IV(%)
sgum	sweetgum	*Liquidambar styraciflua* L.	32.436
pawp	pawpaw	*Asimina triloba* (L.) Dunal	15.688
dhol	deciduous holly	*Ilex decidua* Walter	11.473
ahol	American holly	*Ilex opaca* Aiton	9.980
sugb	sugarberry	*Celtis laevigata* Willd.	5.129
ash	ash	*Fraxinus* spp.	4.331
aelm	American elm	*Ulmus americana* L.	3.591
horn	hornbeam, ironwood	*Carpinus caroliniana* Walter	2.680
boxe	boxelder	*Acer negundo* L.	2.185
laur	laurel oak	*Quercus laurifolia* Michx.	1.545
sycm	sycamore	*Platanus occidentalis* L.	1.180
bitr	bitternut hickory	*Carya cordiformis* (Wang.) K. Koch	1.112
gash	green ash	*Fraxinus pennsylvanica* Marsh	1.083
rmap	red maple	*Acer rubrum* L.	0.981
coak	swamp chestnut oak	*Quercus michauxii* Nutt.	0.914
welm	winged elm	*Ulmus alata* Michx.	0.796
bech	beech	*Fagus grandifolia* Ehrh.	0.755
shok	Shumard oak	*Quercus shumardii* Buckley	0.616
hick	hickory	*Carya* spp.	0.568
spic	spicebush	*Lindera benzoin* (L.) Blume	0.565
hawt	hawthorn	*Crataegus* spp.	0.458
stup	swamp tupelo	*Nyssa biflora* Walter	0.390
ypop	yellow poplar	*Liriodendron tulipifera* L.	0.312
rhik	shagbark hickory	*Carya ovata* (Mill.) K. Koch	0.181
tupe	water tupelo	*Nyssa aquatica* L.	0.177
ocup	overcup oak	*Quercus lyrata* Walter	0.128
unkn	unknown	unknown	0.121
selm	slippery elm	*Ulmus rubra* Muhl.	0.106
cbok	cherrybark oak	*Quercus pagoda* Raf.	0.106
pers	persimmon	*Diospyros virginiana* L.	0.105

whik	water hickory	*Carya aquatica* (Michx. f.) Nutt.	0.086
mulb	red mulberry	*Morus rubra* L.	0.080
plan	water elm	*Planera aquatica* J.F. Gmel.	0.069
rbuc	red buckeye	*Aesculus pavia* L.	0.038
bgum	black gum	*Nyssa sylvatica* Marsh.	0.038
TOTAL IV			100.000

Table S6. ID code, scientific name, and Importance Value (IV) for tree species in Congaree pine hardwood plots.

Species code	Common Name	Scientific Name	IV(%)
horn	hornbeam, ironwood	*Carpinus caroliniana* Walter	27.248
lobp	loblolly pine	*Pinus taeda* L.	26.533
sgum	sweetgum	*Liquidambar styraciflua* L.	11.836
ahol	American holly	*Ilex opaca* Aiton	7.023
rmap	red maple	*Acer rubrum* L.	6.759
wlok	willow oak	*Quercus phellos* L.	5.056
welm	winged elm	*Ulmus alata* Michx.	4.067
laur	laurel oak	*Quercus laurifolia* Michx.	2.231
gash	green ash	*Fraxinus pennsylvanica* Marsh.	1.971
stup	swamp tupelo	*Nyssa biflora* Walter	1.414
pawp	pawpaw	*Asimina triloba* (L.) Dunal	1.321
coak	swamp chestnut oak	*Quercus michauxii* Nutt.	1.305
selm	slippery elm	*Ulmus rubra* Muhl.	0.936
dhol	deciduous holly	*Ilex decidua* Walter	0.550
elm	elm	*Ulmus* spp.	0.426
aelm	American elm	*Ulmus americana* L.	0.399
bgum	black gum	*Nyssa sylvatica* Marsh.	0.363
wsum	winged sumac	*Rhus copallinum* L.	0.200
rbay	redbay	*Persea borbonia* (L.) Spreng	0.183
wash	white ash	*Fraxinus americana* L.	0.181
TOTAL IV			100.000

Table S7. ID code, scientific name, and Importance Value (IV) for tree species in Hobcaw cypress-tupelo vegetation type.

Species code	Common Name	Scientific Name	IV(%)
waxm	wax myrtle	*Morella cerifera* (L.) Small	31.606
lobp	loblolly pine	*Pinus taeda* L.	21.213
bcyp	baldcypress	*Taxodium distichum* (L.) Rich.	18.014
bgum	black gum	*Nyssa sylvatica* Marsh.	9.814
tall	Chinese tallow	*Triadica sebifera* (L.) Small	8.898
rbay	redbay	*Persea borbonia* (L.) Spreng	2.951
laur	laurel oak	*Quercus laurifolia* Michx.	2.312
sgum	sweetgum	*Liquidambar styraciflua* L.	2.071
woak	water oak	*Quercus nigra* L.	0.749
gash	green ash	*Fraxinus pennsylvanica* Marsh.	0.690
tupe	water tupelo	*Nyssa aquatica* L.	0.572
butn	buttonbush	*Cephalanthus occidentalis* L.	0.187
unkn	unknown	unknown	0.183
selm	slippery elm	*Ulmus rubra* Muhl.	0.117
blue	blueberry	*Vaccinium elliottii* Chapm.	0.116
wash	white ash	*Fraxinus americana* L.	0.116
ahol	American holly	*Ilex opaca* Aiton	0.083
pers	persimmon	*Diospyros virginiana* L.	0.081
dahn	dahoon holly	*Ilex cassine* L.	0.064
rmap	red maple	*Acer rubrum* L.	0.060
will	black willow	*Salix nigra* Marsh.	0.042
yaup	yaupon	*Ilex vomitoria* Aiton	0.036
aelm	American elm	*Ulmus americana* L.	0.012
inkb	inkberry	*Ilex glabra* (L.) A. Gray	0.011
TOTAL IV			100.000

Table S8. ID code, scientific name, and Importance Value (IV) for tree species in Hobcaw upland pine hardwood vegetation type.

Species code	Common Name	Scientific Name	IV(%)
lobp	loblolly pine	*Pinus taeda* L.	77.008
waxm	wax myrtle	*Morella cerifera* (L.) Small	5.833
live	live oak	*Quercus virginiana* Mill.	5.198
sgum	sweetgum	*Liquidambar styraciflua* L.	3.440
pndp	pond pine	*Pinus serotina* Michx.	2.121
rbay	redbay	*Persea borbonia* (L.) Spreng	2.072
yaup	yaupon	*Ilex vomitoria* Aiton	1.521
tall	Chinese tallow	*Triadica sebifera* (L.) Small	0.897
hsug	horse sugar	*Symplocos tinctoria* (L.) L'Hér.	0.679
laur	laurel oak	*Quercus laurifolia* Michx.	0.504
bgum	black gum	*Nyssa sylvatica* Marsh.	0.309
woak	water oak	*Quercus nigra* L.	0.116
blue	blueberry	*Vaccinium elliottii* Chapm.	0.111
ahol	American holly	*Ilex opaca* Aiton	0.103
poak	post oak	*Quercus stellata* Wangenh.	0.036
inkb	inkberry	*Ilex glabra* (L.) A. Gray	0.024
wlok	willow oak	*Quercus phellos* L.	0.012
dhol	deciduous holly	*Ilex decidua* Walter	0.008
unkn	unknown	unknown	0.008
TOTAL IV			100.000

Table S9. ID code, scientific name, and Importance Value (IV) for tree species in Santee cypress-tupelo vegetation type.

Species code	Common Name	Scientific Name	IV(%)
bcyp	baldcypress	*Taxodium distichum* (L.) Rich.	16.804
laur	laurel oak	*Quercus laurifolia* Michx.	15.149
horn	hornbeam, ironwood	*Carpinus caroliniana* Walter	12.601
gash	green ash	*Fraxinus pennsylvanica* Marsh.	11.701
sgum	sweetgum	*Liquidambar styraciflua* L.	7.348
rmap	red maple	*Acer rubrum* L.	5.887
ahol	American holly	*Ilex opaca* Aiton	4.580
dhol	deciduous holly	*Ilex decidua* Walter	3.643
whik	water hickory	*Carya aquatica* (Michx. f.) Nutt	2.629
ocup	overcup oak	*Quercus lyrata* Walte	2.627
aelm	American elm	*Ulmus americana* L.	2.297
coak	swamp chestnut oak	*Quercus michauxii* Nutt.	1.677
vibo	viburnum	*Viburnum* spp	1.600
selm	slippery elm	*Ulmus rubra* Muhl.	1.194
bgum	black gum	*Nyssa sylvatica* Marsh.	1.149
welm	winged elm	*Ulmus alata* Michx	1.143
tall	Chinese tallow	*Triadica sebifera* (L.) Small	1.091
lobp	loblolly pine	*Pinus taeda* L.	1.091
unkn	unknown	unknown	1.022
will	black willow	*Salix nigra* Marsh.	0.896
sugb	sugarberry	*Celtis laevigata* Willd.	0.773
sdog	swamp dogwood	*Cornus foemina* Mill.	0.764
woak	water oak	*Quercus nigra* L.	0.738
butn	buttonbush	*Cephalanthus occidentalis* L.	0.633
hawt	hawthorn	*Crataegus* spp.	0.225
cbok	cherrybark oak	*Quercus pagoda* Raf.	0.211
mhik	mockernut hickory	*Carya tomentosa* (Lam.) Nutt.	0.185
mulb	red mulberry	*Morus rubra* L.	0.054
wsum	winged sumac	*Rhus copallinum* L.	0.040
elm	elm	*Ulmus* spp.	0.036

wlok	willow oak	*Quercus phellos* L.	0.035
blko	black oak	*Quercus velutina* Lam.	0.034
cwil	coastal plains willow	*Salix caroliniana* Michx.	0.022
pers	persimmon	*Diospyros virginiana* L.	0.020
rbay	redbay	*Persea borbonia* (L.) Spreng	0.018
live	live oak	*Quercus virginiana* Mill.	0.017
shok	Shumard oak	*Quercus shumardii* Buckley	0.017
cedr	eastern redcedar	*Juniperus virginiana* L.	0.017
ypop	yellow poplar	*Liriodendron tulipifera* L.	0.016
waxm	wax myrtle	*Morella cerifera* (L.) Small	0.016
TOTAL IV			100.000

Table S10. ID code, scientific name, and Importance Value (IV) for tree species in Santee bottomland hardwood vegetation type.

Species code	Common Name	Scientific Name	IV(%)
rmap	red maple	*Acer rubrum* L.	28.074
laur	laurel oak	*Quercus laurifolia* Michx.	20.873
sgum	sweetgum	*Liquidambar styraciflua* L.	10.499
horn	ironwood	*Carpinus caroliniana* Walter	8.329
selm	slippery elm	*Ulmus rubra* Muhl.	6.633
gash	green ash	*Fraxinus pennsylvanica* Marsh.	4.646
wlok	willow oak	*Quercus phellos* L.	2.946
bgum	black gum	*Nyssa sylvatica* Marsh.	2.812
dhol	deciduous holly	*Ilex decidua* Walter	2.416
welm	winged elm	*Ulmus alata* Michx	1.972
waxm	wax myrtle	*Morella cerifera* (L.) Sma	1.809
sdog	swamp dogwood	*Cornus foemina* Mill.	1.634
blko	swamp dogwood	*Cornus foemina* Mill.	1.366
coak	swamp chestnut oak	*Quercus michauxii* Nutt.	1.126
ahol	American holly	*Ilex opaca* Aiton	1.111
whik	water hickory	*Carya aquatica* (Michx. f.) Nutt.	0.693
pers	persimmon	*Diospyros virginiana* L.	0.665
unkn	unknown	unknown	0.579
bcyp	baldcypress	*Taxodium distichum* (L.) Rich.	0.497
swill	black willow	*Salix nigra* Marsh.	0.275
dogw	flowering dogwood	*Cornus florida* L.	0.192
tall	Chinese tallow	*Triadica sebifera* (L.) Small	0.151
vibo	viburnum	*Viburnum* spp.	0.127
lobp	loblolly pine	*Pinus taeda* L.	0.126
sugb	sugarberry	*Celtis laevigata* Willd.	0.112
hsug	horse sugar	*Symplocos tinctoria* (L.) L'Hér.	0.093
woak	water oak	*Quercus nigra* L.	0.080
rhik	shagbark hickory	*Carya ovata* (Mill.) K. Koch	0.065
aelm	American elm	*Ulmus americana* L.	0.054
tupe	water tupelo	*Nyssa aquatica* L.	0.015

butn	buttonbush	*Cephalanthus occidentalis* L.	0.014
hawt	hawthorn	*Crataegus spp.*	0.140
TOTAL IV			100.000

Table S11. ID code, scientific name, and Importance Value (IV) for tree species in Santee upland pine hardwood vegetation type.

Species code	Common Name	Scientific Name	IV(%)
lobp	loblolly pine	*Pinus taeda* L.	48.354
woak	water oak	*Quercus nigra* L.	13.885
bgum	black gum	*Nyssa sylvatica* Marsh.	11.188
sgum	sweetgum	*Liquidambar styraciflua* L.	8.244
rmap	red maple	*Acer rubrum* L.	6.709
rbay	redbay	*Persea borbonia* (L.) Spreng	2.385
poak	post oak	*Quercus stellata* Wangenh.	2.316
ahol	American holly	*Ilex opaca* Aiton	2.135
hsug	horse sugar	*Symplocos tinctoria* (L.) L'Hér.	1.290
wlok	willow oak	*Quercus phellos* L.	1.038
waxm	wax myrtle	*Morella cerifera* (L.) Small	0.988
blue	blueberry	*Vaccinium elliottii* Chapm.	0.367
long	longleaf pine	*Pinus palustris* Mill.	0.185
blko	black oak	*Quercus velutina* Lam.	0.136
laur	laurel oak	*Quercus laurifolia* Michx.	0.136
shrt	shortleaf pine	*Pinus echinata* Mill.	0.118
live	live oak	*Quercus virginiana* Mill.	0.111
sred	southern red oak	*Quercus falcata* Michx.	0.090
sbay	sweetbay	*Magnolia virginiana* L.	0.088
bech	beech	*Fagus grandifolia* Ehrh.	0.049
welm	winged elm	*Ulmus alata* Michx.	0.033
unkn	unknown	unknown	0.024
dogw	flowering dogwood	*Cornus florida* L.	0.024
cher	black cherry	*Prunus serotina* Ehrh.	0.023
wsum	winged sumac	*Rhus copallinum* L.	0.023
cbok	cherrybark oak	*Quercus pagoda* Raf.	0.014
gash	green ash	*Fraxinus pennsylvanica* Marsh.	0.013
unkn2	unknown2	unknown2	0.012
bjok	blackjack oak	*Quercus marilandica* Münchh.	0.012
dlau	dwarf laurel	*Prunus laurocerasus*	0.011
TOTAL IV			100.000

Supplementary data
Data trends

Data trends from the entire study period to date (1994-2016) are presented here in order to display long-term changes (**Figs. S1-S11)**. Please note that although the format of the figures is uniform, the scale changes greatly due to varying overstory types.

Beidler Forest

Cypress-tupelo plots were dominated in 1994 by bald cypress (*Taxodium distichum* (L.) Rich., 29.3 m^2/ha, 103.8 stems/ha) and water tupelo (*Nyssa aquatica* L.*,* 35.9 m^2/ha, 286.3 stems/ha) trees with a smaller black gum (*Nyssa sylvatica* Marsh.) component. The sapling layer was almost exclusively made up of green ash (*Fraxinus pennsylvanica* Marsh.) throughout the study period. Sapling-size green ash stems decreased 30% from 1994 to 2016 (1248.8 to 875 stems/ha) but remained relatively high. The density of green ash tree-size stems increased steadily by 36% from 1994 to 2016. Bald cypress, tupelo, and black gum BA and density changed little in both trees and saplings (**Fig. S1**).

The bottomland hardwoods were dominated in 1994 (the first year of sampling) by sweetgum (*Liquidambar styraciflua* L., 9.5 m^2/ha, 50 stems/ha), laurel oak (*Quercus laurifolia* Michx., 7.3 m^2/ha, 211.3 stems/ha), and bald cypress (5.6 m^2/ha, 53.8 stems/ha). The sapling layer consisted of laurel oak (215 stems/ha), hornbeam (*Carpinus caroliniana* Walt., 143.8 stems/ha), green ash (85 stems/ha), and red maple (*Acer rubrum* L., 83.8 stems/ha). Tree-size bald cypress and sweetgum BA increased slightly (**Fig. S2**). Laurel oak trees increased in BA throughout the study and have assumed a dominant position in the overstory. Tree-size laurel oak were the most abundant throughout the study period. In the sapling layer, the dominant laurel oak, hornbeam, and red maple numbers were steady until 2000 but have declined since then (**Fig. S2**).

In 1994, the ridge bottom plots contained a diverse overstory of sweetgum (2.4 m^2/ha), water oak (*Quercus nigra* L.*,* 1.7 m^2/ha), hornbeam (1.3 m^2/ha), and laurel oak (0.43 m^2/ha). Sweetgum, water oak, hornbeam, and laurel oak trees increased in BA and density up to 2016. The most dominant tree was hornbeam (**Fig. S3**) whose density doubled between 1994 and 2000. In 2016 hornbeam remained the most abundant while the less-dense sweetgum had the highest BA (85 stems/ha, 4.5 m^2/ha). The sapling layer was mostly hornbeam in 1994 but included sweetgum, water oak, American holly (*Ilex opaca* Ait.), and laurel oak. Hornbeam remained dominant in the sapling layer throughout the study (**Fig. S3**). The other species remained mostly unchanged by 2016, except for American holly which mildly increased in both BA and density consistently from 1994 (**Fig. S3**).

A.

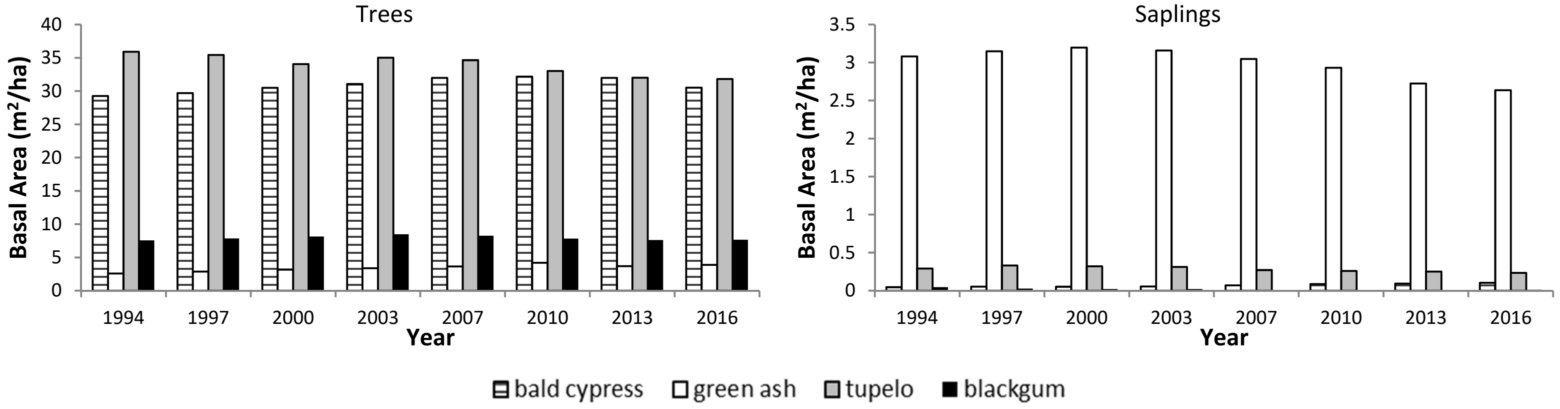

B.

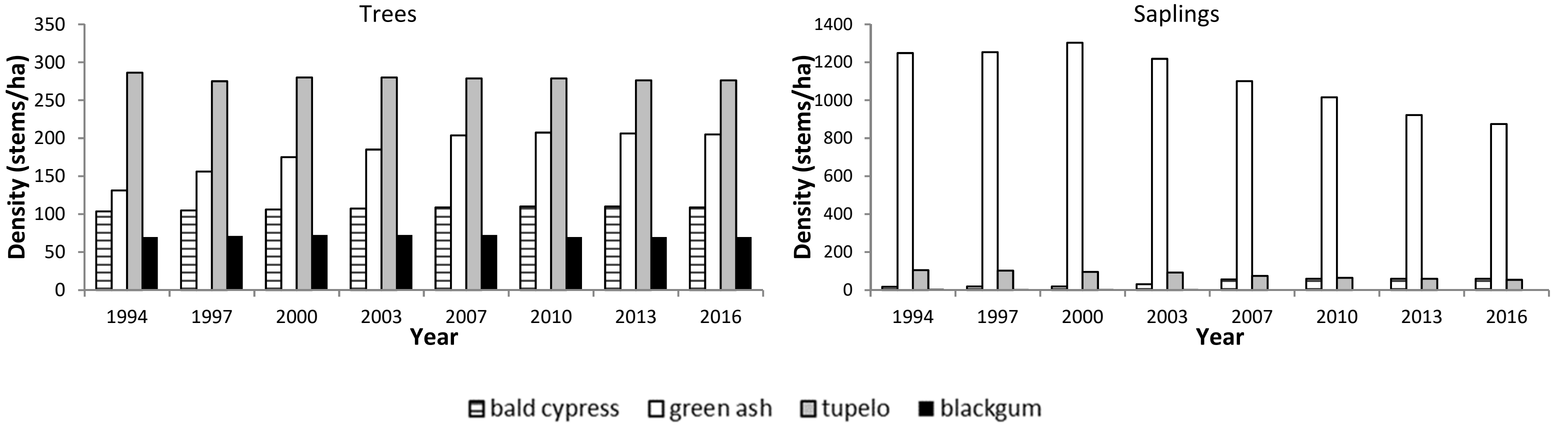

Figure S1 (A) Basal area and (B) density changes for dominant species in the cypress-tupelo type at Beidler Forest.

A.

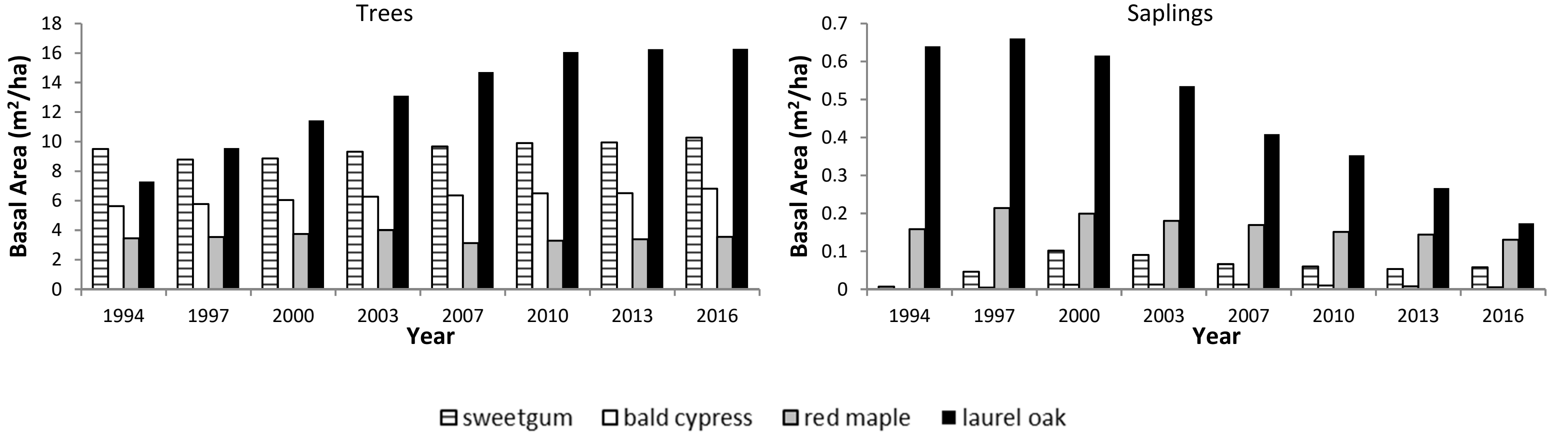

B.

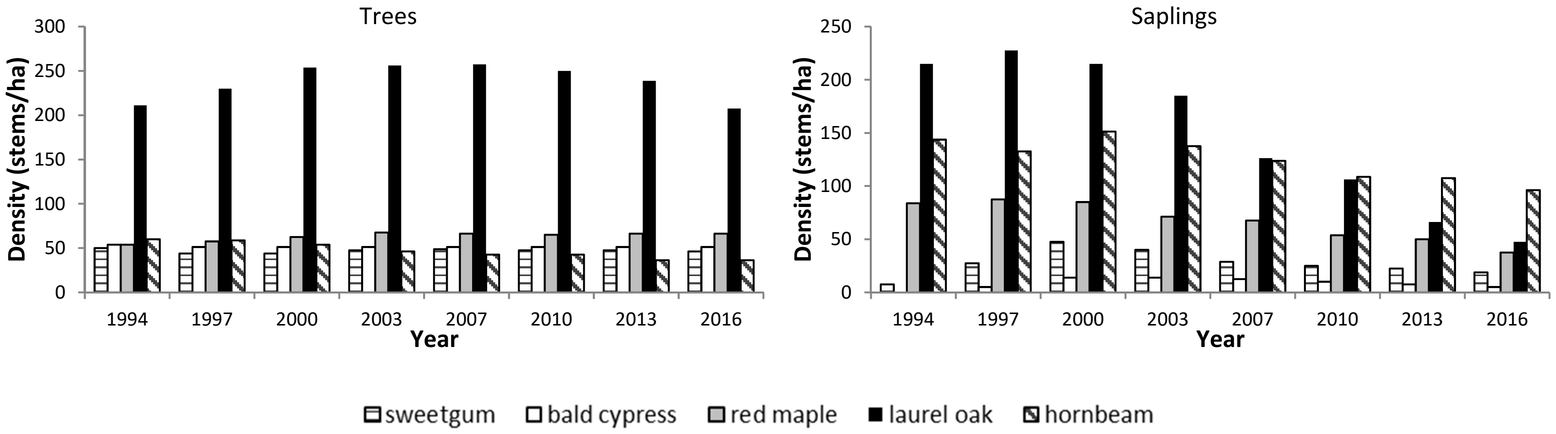

Figure S2 (**A**) Basal area and (**B**) density changes for dominant species in the bottomland hardwood forest type at Beidler Forest.

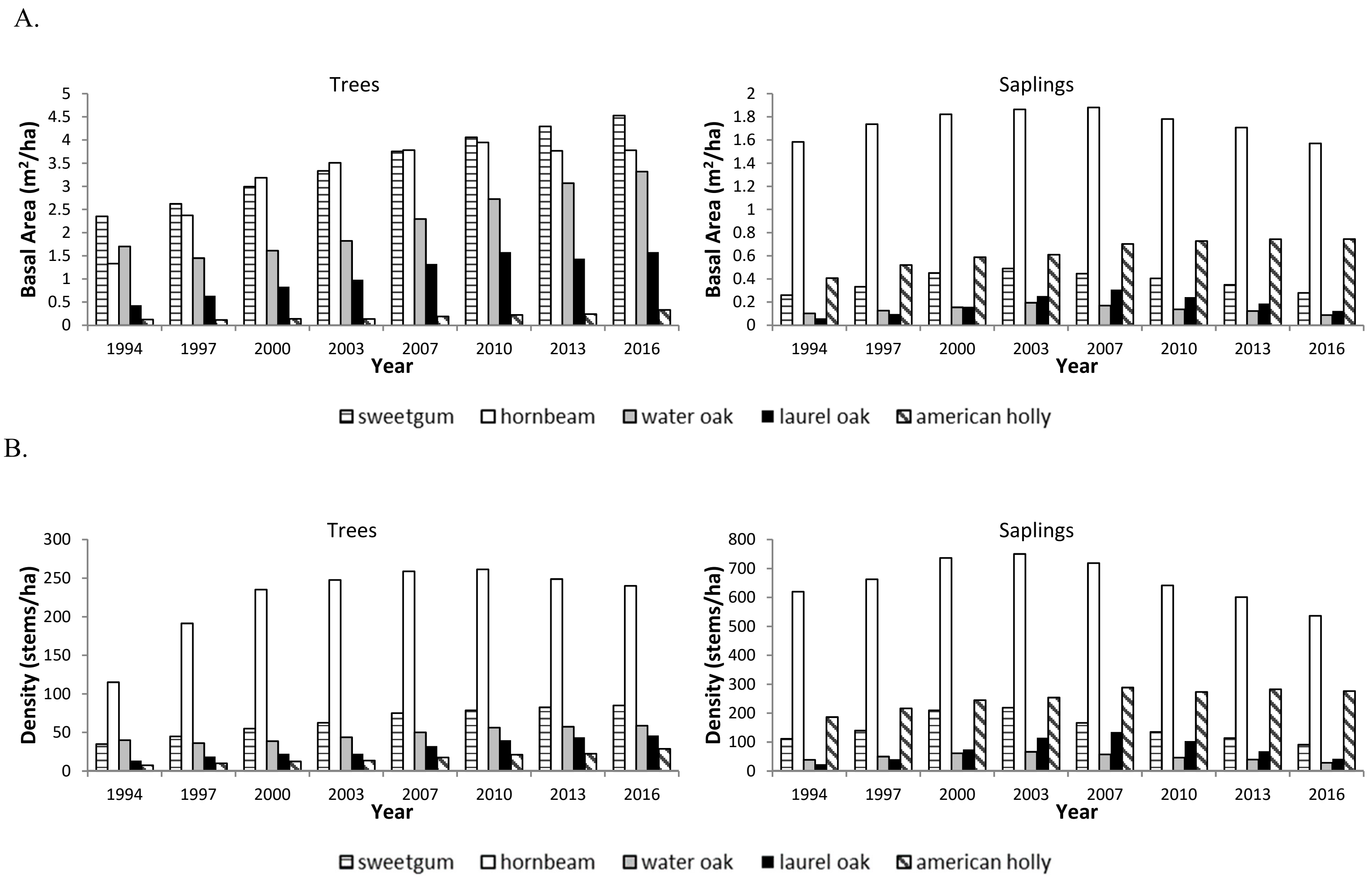

Figure S3 (**A)** Basal area and (**B**) density changes for dominant species in the ridge bottom forest type at Beidler Forest.

Congaree National Park

The cypress-tupelo forest type plots were dominated in 1994 in the tree size class by water tupelo (27.3 m^2/ha, 298.8 stems/ha), bald cypress (14.3 m^2/ha, 91.3 stems/ha) and swamp tupelo (*Nyssa biflora* Walt., 9.1 m^2/ha, 101.3 stems/ha). Most of the 1994 sapling strata consisted of white ash (*Fraxinus americana* L., 130 stems/ha) and water tupelo (40 stems/ha). Water tupelo, bald cypress, and swamp tupelo trees increased in BA during the study period (**Fig. S4**), while other species changed little in BA. Red maple sapling density increased nearly five-fold by 2007, then decreased by about 31% by 2016. The density of white ash saplings decreased throughout the study period, resulting in a 2016 population of less than half of the 1994 population (**Fig. S4**).

In 1994, the bottomland hardwood plots were dominated by sweetgum (25.4 m^2/ha) and to a lesser extent American holly (2.8 m^2/ha). The sapling layer was initially dominated by deciduous holly (*Ilex decidua* Walt., 0.23 m^2/ha, 109 stems/ha) and pawpaw (*Asimina triloba* (L.) Dunal, 0.11 m^2/ha, 99 stems/ha). Deciduous holly, American holly, and pawpaw sapling density increased dramatically between the 2003 and 2007 censuses. The BA of sweetgum and American holly trees changed little throughout the study (**Fig. S5**).

The pine-hardwood plot of Congaree was dominated in 1994 in the tree strata by loblolly pine (Pinus taeda L., 17.7 m^2/ha, 35 stems/ha), sweetgum (6.9 m^2/ha, 50 stems/ha), and hornbeam (2.6 m^2/ha, 150 stems/ha). The sapling layer was established in 2007 and was mostly hornbeam (0.93 m^2/ha, 335 stems/ha), American holly (0.34 m^2/ha, 115 stems/ha), and red maple (0.19 m^2/ha, 75 stems/ha). Tree-size loblolly pine BA increased slightly by 2016 and the density of tree-size loblolly pine remained consistent (**Fig. S6**). Hornbeam was the most abundant in the tree layer and in the sapling layer throughout the study.

A.

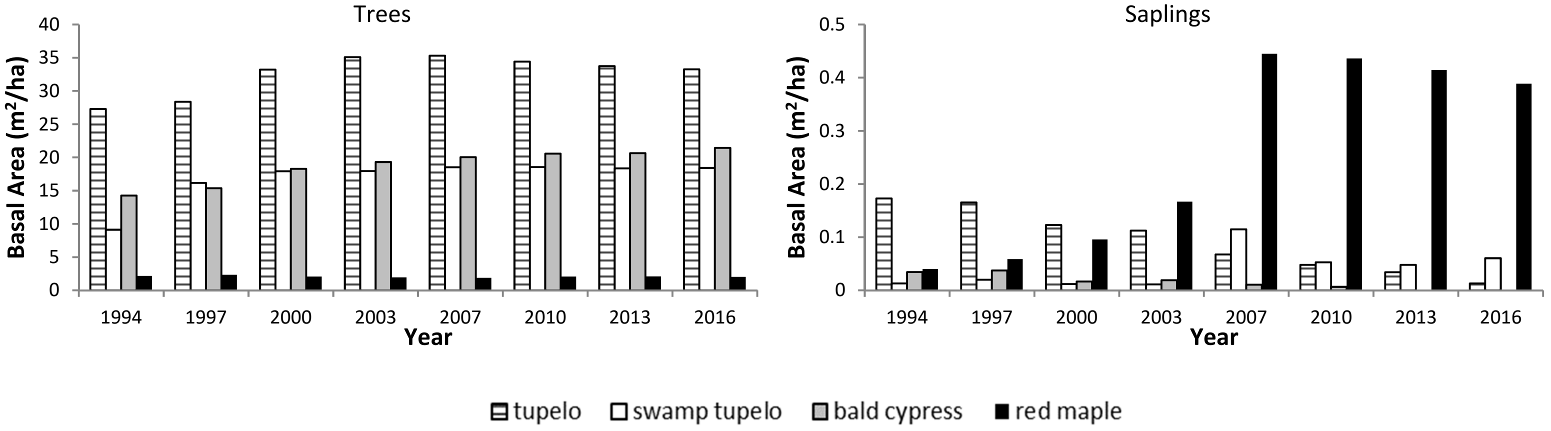

B.

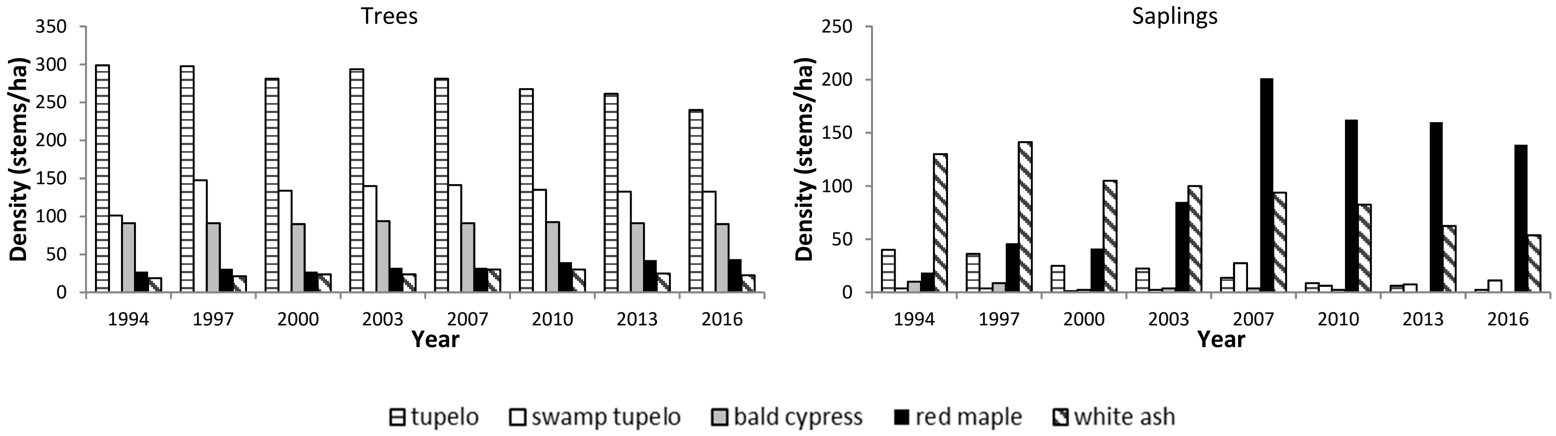

Figure S4 (**A**) Basal area and (**B**) density changes for important species in the cypress-tupelo forest type at Congaree National Park.

A.

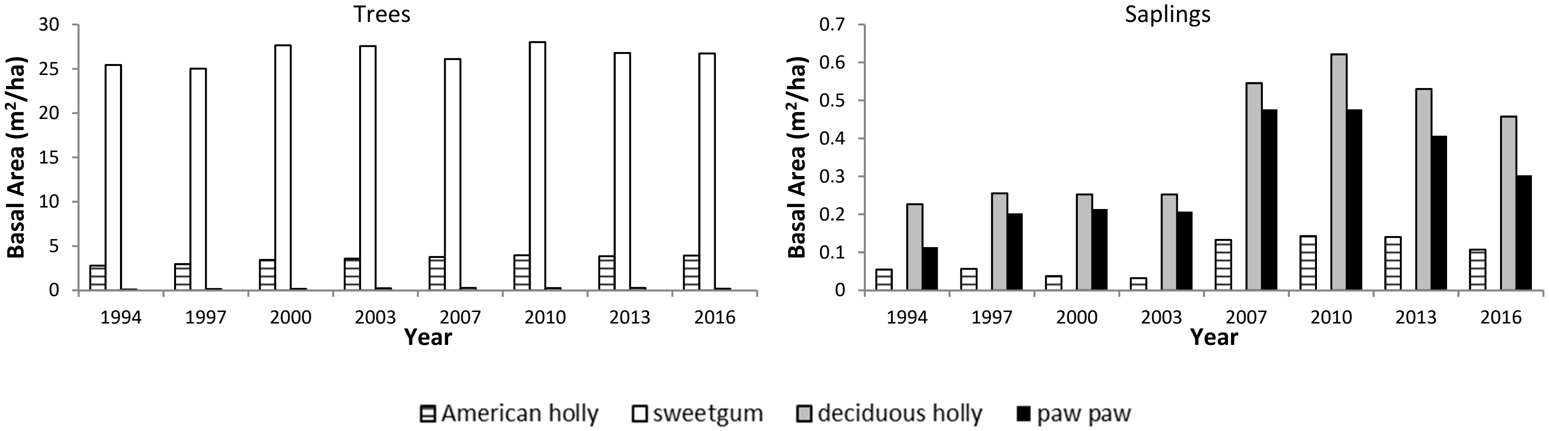

B.

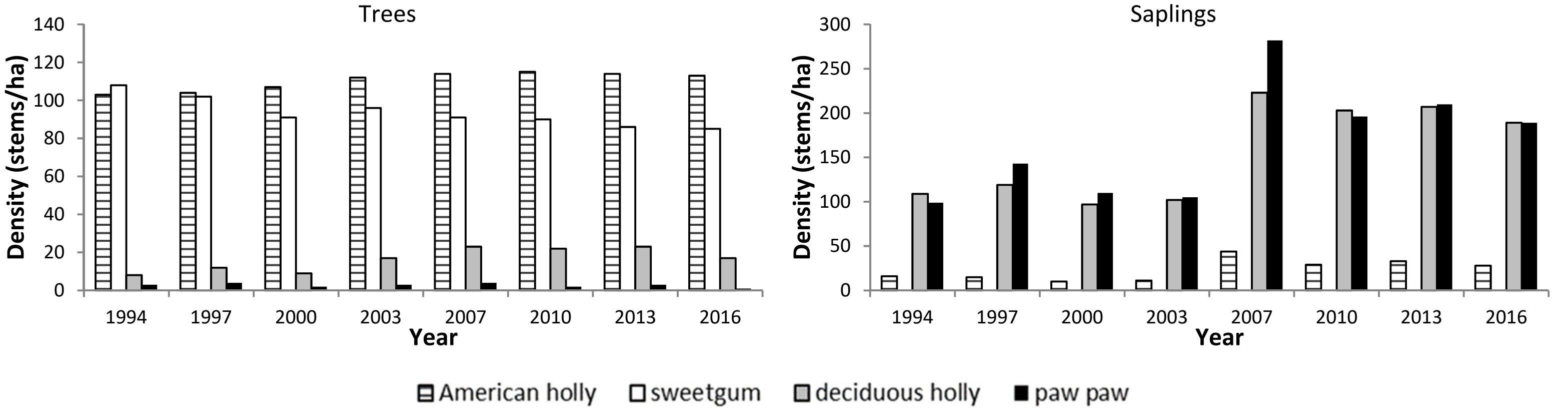

Figure S5 (**A**) Basal area and (**B**) density changes for important species in the bottomland hardwood forest type at Congaree National Park.

A.

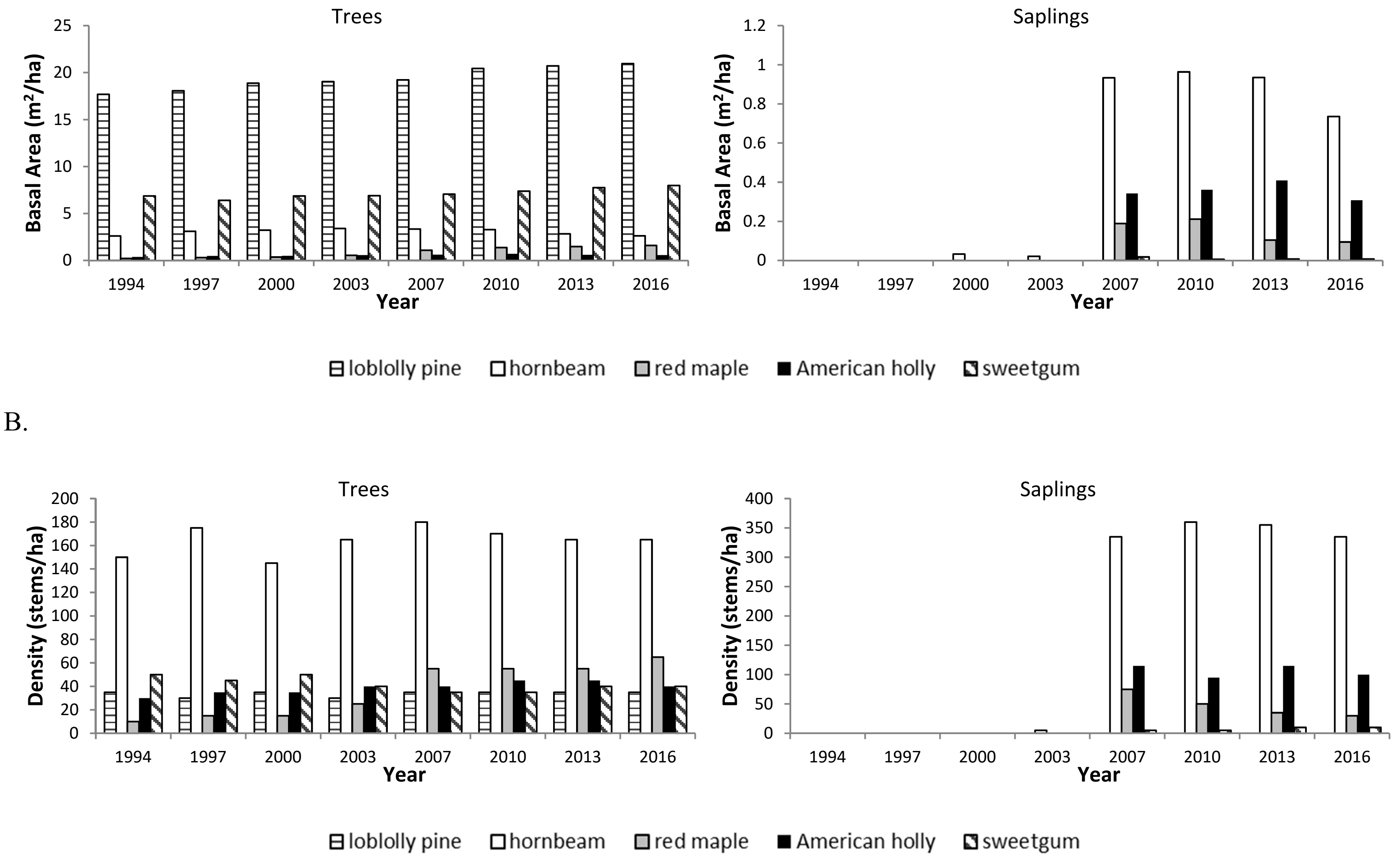

Figure S6 (**A**) Basal area and (**B**) density changes for important species in the pine hardwood forest type at Congaree National Park.

Hobcaw Barony

In 1994, the tree strata of the cypress-tupelo plots consisted of black gum (4.3 m^2/ha, 83.8 stems/ha), bald cypress (3.8 m^2/ha, 45 stems/ha), and laurel oak (1.1 m^2/ha, 7.5 stems/ha). Most sapling-sized stems were wax myrtle (*Morella cerifera* (L.) Small, 250 stems/ha), loblolly pine (46.3 stems/ha), and redbay (*Persea borbonia* (L.) Spreng., 33.8 stems/ha). Black gum tree BA and density remained consistent throughout the study period. Loblolly pine BA increased to about one hundred and twelve times the initial amount by 2016 and loblolly density increased as well (**Fig. S7**), surpassing black gum in both tree BA and density. Bald cypress tree density nearly quadrupled over the 23-year period and Chinese tallow density increased thirty-two times from 1997 to 2016, while other tree densities were relatively unchanged (**Fig. S7**). The number of wax myrtle stems entering the tree layer was low throughout the study and by 2016 wax myrtle tree density reached 22.5 stems/ha. In the sapling strata, wax myrtle density rose substantially from 250 stems/ha in 1994 to 2,195 stems/ha in 2007, then decreased to 1,277.5 stems/ha by 2016 (**Fig. S7**). Bald cypress, loblolly, and red bay sapling densities also increased, resulting in a dense sapling layer by the end of the study period.

Both the tree and the sapling layer in upland pine-hardwood plots were dominated by loblolly pine throughout the study. Live oak tree-size stems increased by 115% in BA and 80% in density over the study period. Wax myrtle and red bay represented a smaller component of the sapling layer and had slight growth in density and BA from 1994 to 2016. (**Fig. S8**)

A.

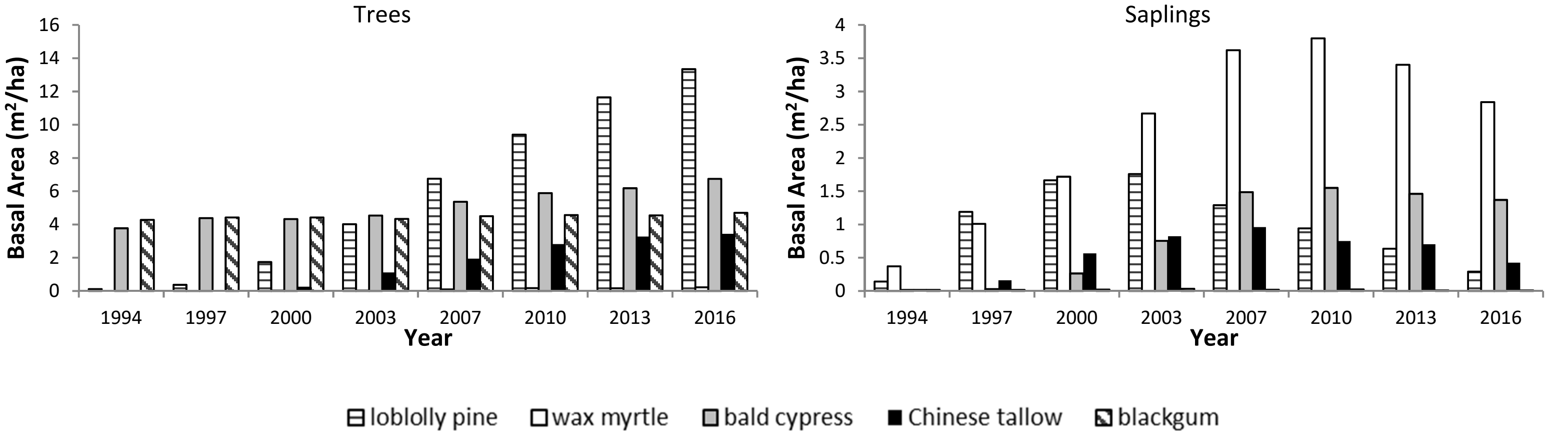

B.

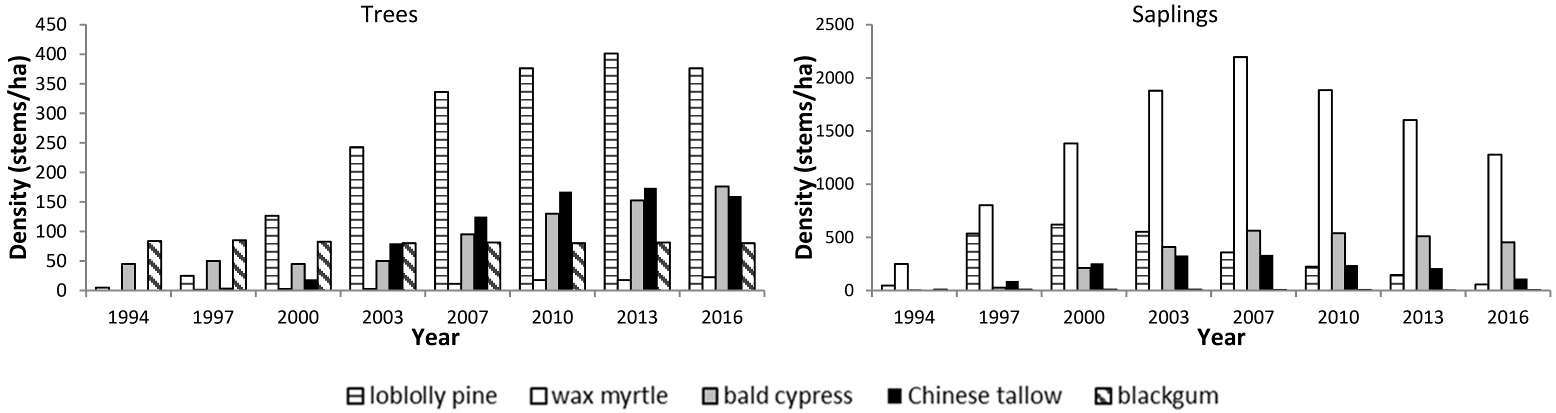

Figure S7 (**A**) Basal area and (**B**) density changes for important species in the cypress-tupelo forest type at Hobcaw Barony.

A.

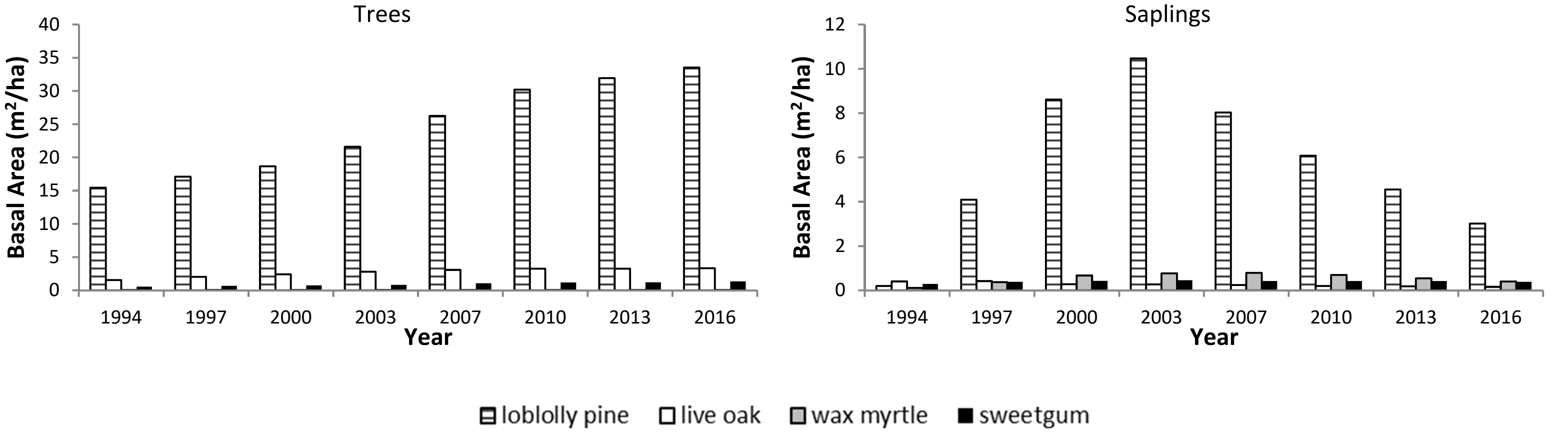

B.

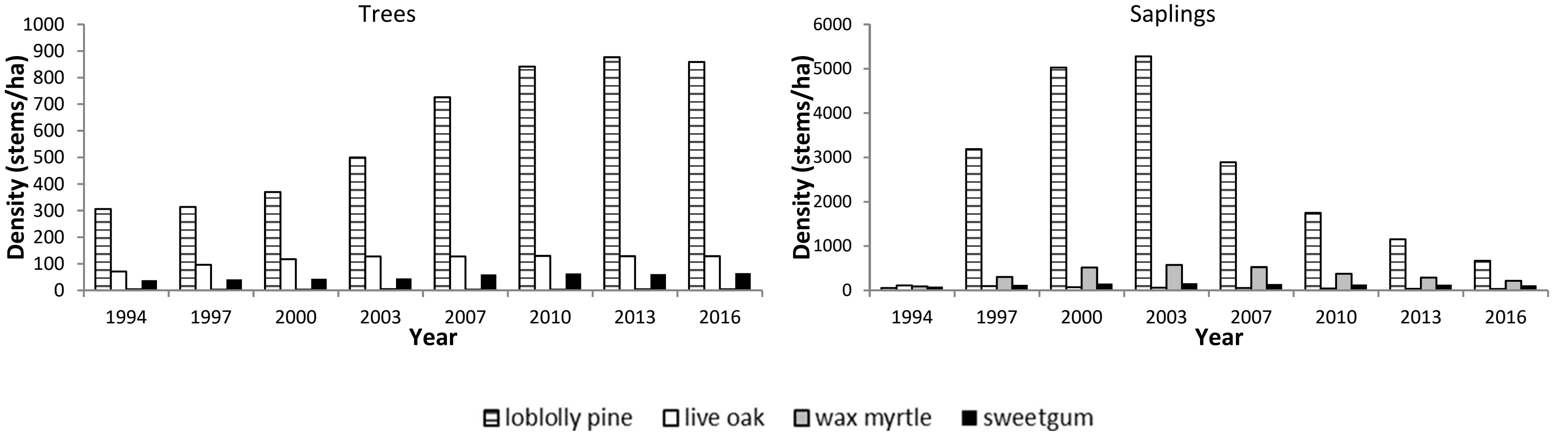

Figure S8 (**A**) Basal area and (**B**) density changes for important species in the upland pine-hardwood forest type at Hobcaw Barony.

Santee Experimental Forest

The cypress-tupelo type plots in Santee were adjacent to the BLH plots and were divided by Nicholson Creek. In 1994, bald cypress contributed 9.7 m^2/ha (30 stems/ha) to the cypress-tupelo forest type (total of 22.3 m^2/ha in BA). Other less-dominant overstory species included sweetgum (2.7 m^2/ha, 11.3 stems/ha), loblolly pine (0.58 m^2/ha, 1.3 stems/ha), and overcup oak (*Quercus lyrata* Walt., 1.2 m^2/ha, 5 stems/ha). Sapling strata consisted of laurel oak (222.5 stems/ha), green ash (187.5 stems/ha), hornbeam (170 stems/ha), and American holly (97.5 stems/ha). The BA and density of laurel oak trees increased substantially over the course of the study, while other species changed little in BA and density (**Fig. S9**). In the sapling strata, green ash and hornbeam increased greatly. Green ash density almost tripled and hornbeam density more than doubled by 2003, but had begun to decline by 2016. American holly sapling density nearly doubled by 2013, but tree BA of the species saw a slight decline throughout the study. Other species in the sapling strata did not increase greatly in density.

The bottomland hardwood plots were dominated in 1994 by red maple (3.7 m^2/ha, 160 stems/ha), laurel oak (1.5 m^2/ha, 21.3 stems/ha), and sweetgum (2.9 m^2/ha, 75 stems/ha) tree-size stems. The BA of red maple, laurel oak, and sweetgum increased with each census (**Fig. S10**). Red maple and sweetgum BA doubled, and laurel oak BA was over five times the initial BA in 2016. Red maple saplings reached a peak density (880 stems/ha) at the 2000 census and laurel oak saplings were at peak density (598.3 stems/ha) in 2003. By 2016 red maple and laurel oak sapling density had decreased by 56% and 43%, respectively. Between 2000 and 2016, red maple and laurel oak tree densities increased by 24% and 133%, respectively. Hornbeam sapling BA and density increased throughout the study, and hornbeam remained a minor component of the tree layer (**Fig. S10**).

The tree layer of the upland pine-hardwood plots mostly consisted of loblolly pine (4.9 m^2/ha, 70 stems/ha), black gum (1.5 m^2/ha, 105 stems/ha), and water oak (1.1 m^2/ha, 66.3 stems/ha) in 1994. Tree-size loblolly pine BA and density increased substantially throughout the study (**Fig. S11**). Black gum and water oak had less dramatic increases in size and abundance and were a much smaller component of the tree layer compared to loblolly pine by 2016. Sapling-size loblolly pine BA and density rose rapidly, reached a peak in 2003, and subsequently decreased by 77% by 2016. Loblolly pine tree density increased by 87% between 2003 and 2016. Water oak saplings followed a similar trend to loblolly saplings but with less dramatic changes in BA and density. Red maple saplings reached a peak density in 2000 (335 stems/ha) and decreased slightly in the following years, while the density of tree-size red maple increased (**Fig. S11**).

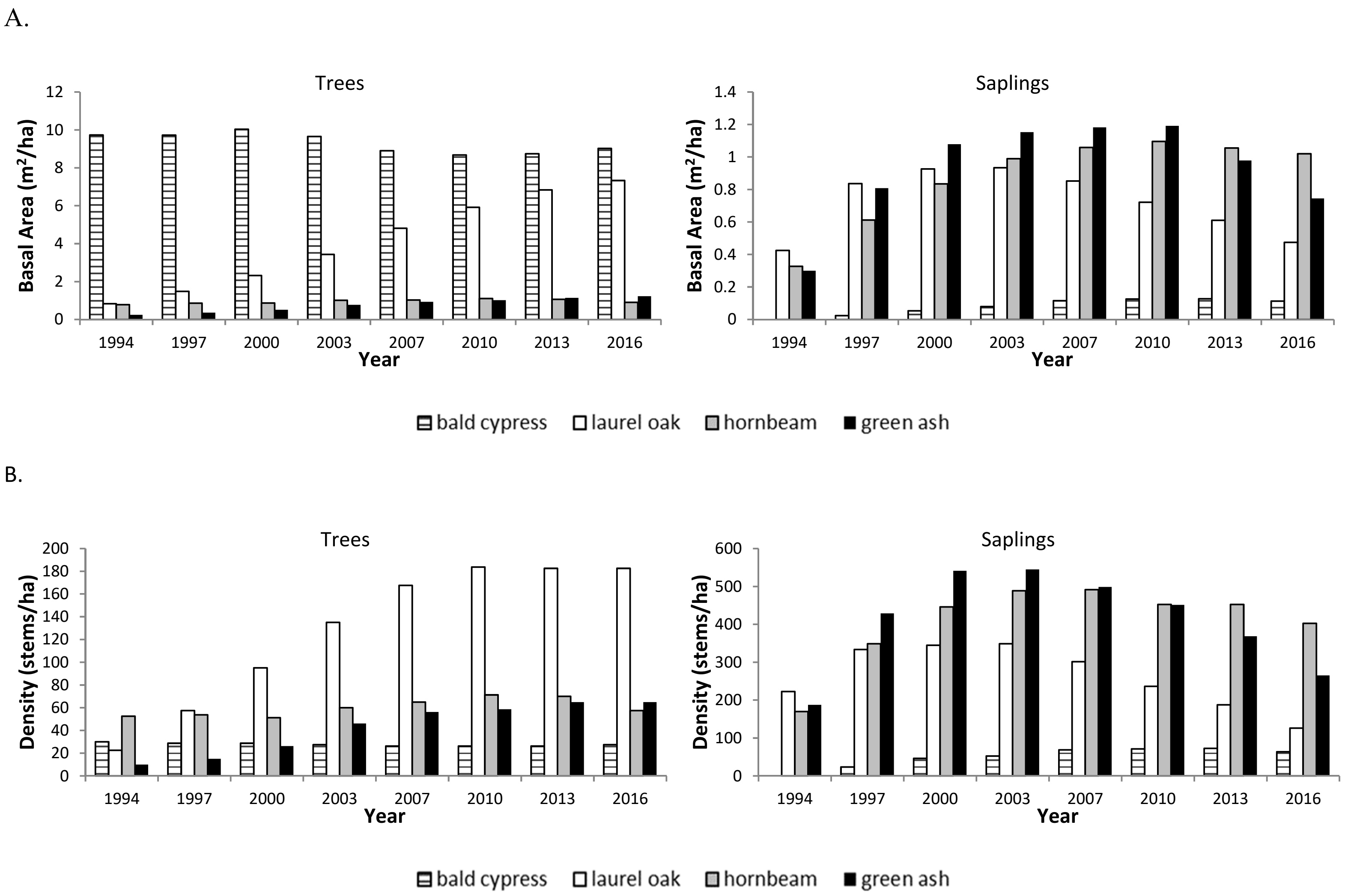

Figure S9 (**A**) Basal area and (**B**) density changes for important species in the cypress-tupelo forest type at Santee Experimental Forest.

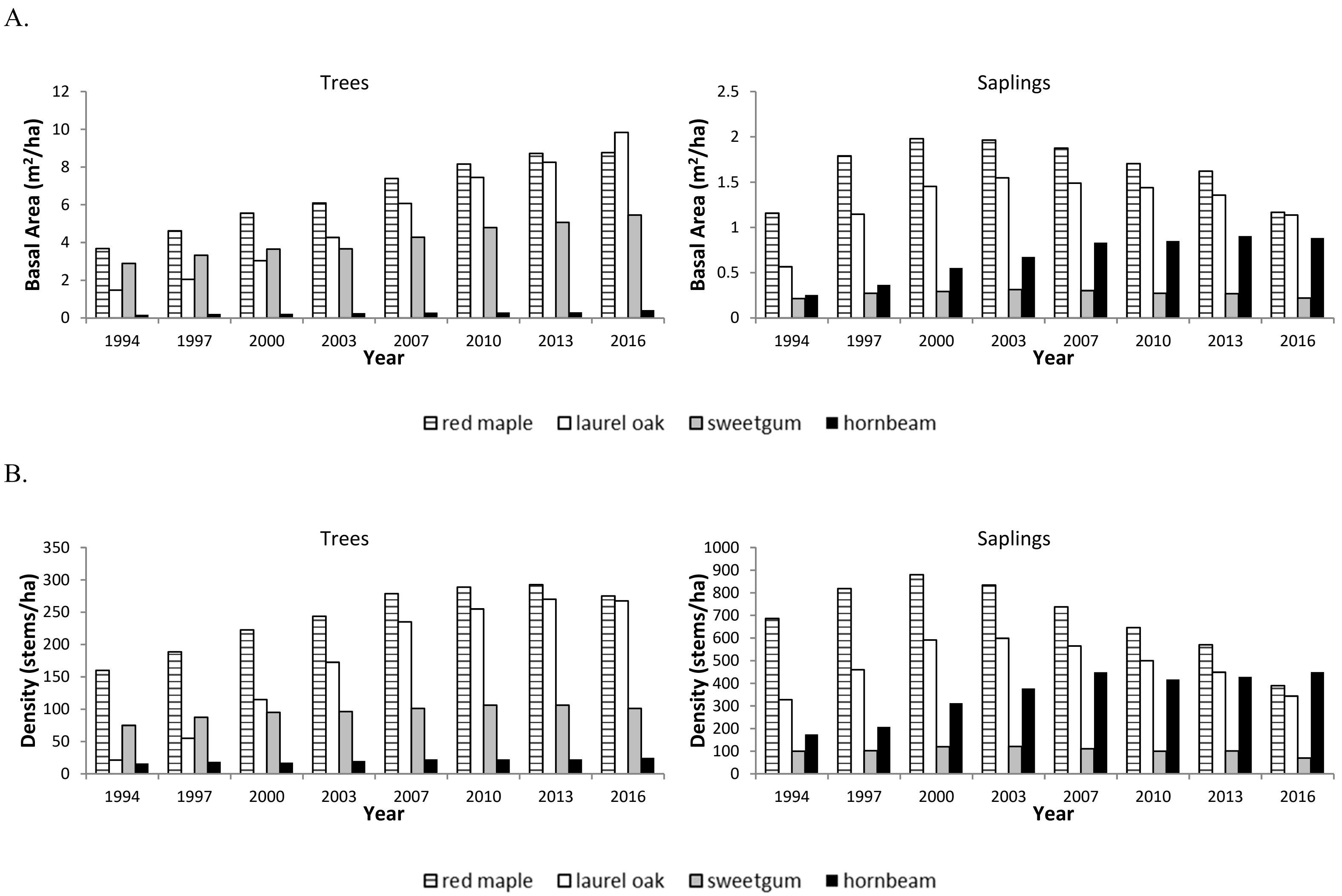

Figure S10 (**A**) Basal area and (**B**) density changes for important species in the bottomland hardwood forest type at Santee Experimental Forest.

A.

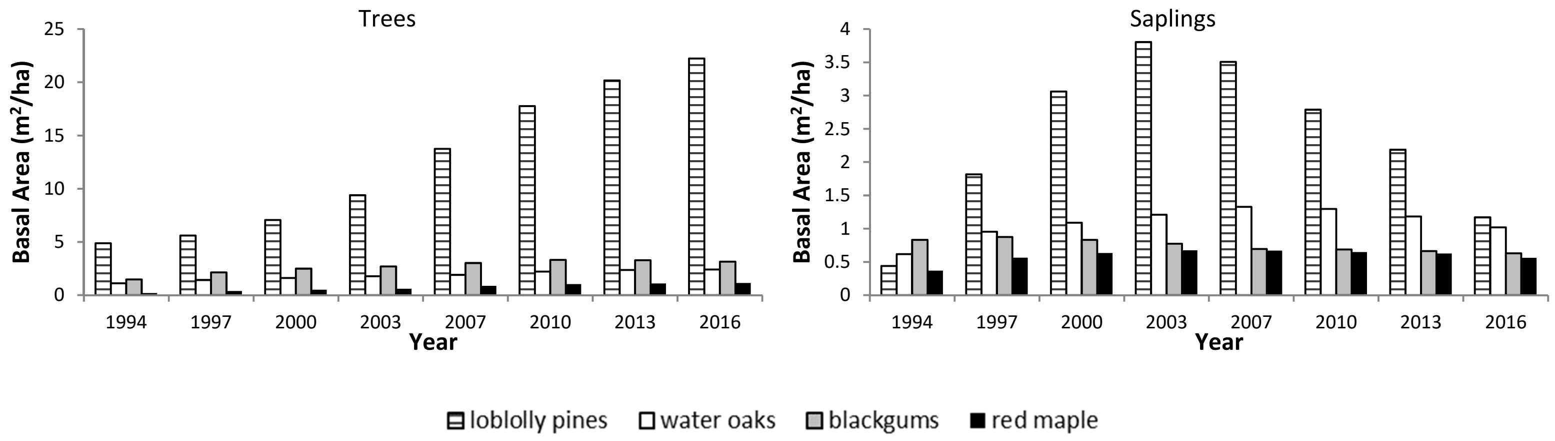

B.

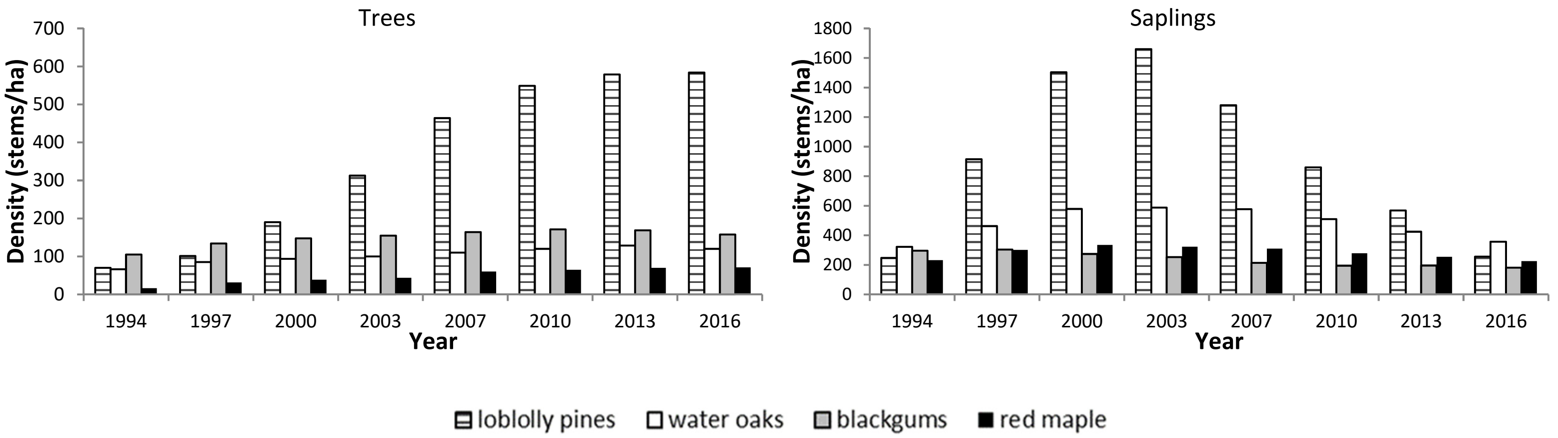

Figure S11 (**A**) Basal area and (**B**) density changes for important species in the upland pine-hardwood forest type at Santee Experimental Forest.
